# Localized delivery of hyaluronic acid-doxorubicin from a surgical paste for post-operative glioblastoma treatment

**DOI:** 10.1016/j.mtbio.2026.103142

**Published:** 2026-04-22

**Authors:** Giulia Rodella, Cristiano Pesce, Riccardo Rampado, Mariangela Garofalo, Mingchao Wang, Bernard Ucakar, Kevin Vanvarenberg, Zhanjun Ma, Nicolas Joudiou, Véronique Préat, Bernard Gallez, Alessio Malfanti

**Affiliations:** a UCLouvain, Louvain Drug Research Institute, Advanced Drug Delivery and Biomaterials, Avenue Mounier 73 B1.73.12, Brussels, 1200, Belgium; b UCLouvain, Louvain Drug Research Institute, Biomedical Magnetic Resonance, Avenue Mounier 73 B1.73.08, Brussels, 1200, Belgium; c Department of Pharmaceutical and Pharmacological Sciences, University of Padova, Via F. Marzolo, 5, Padova, 35131, Italy

**Keywords:** Glioblastoma, Hyaluronic acid, Doxorubicin, Paste scaffold, Local treatment, Nanomedicines, Immunomodulation, Glioblastoma stem cells, Resection

## Abstract

Glioblastoma stem cells (GSCs) and residual tumor cells - which resist conventional therapies, drive disease recurrence, and contribute to the formation of an immunosuppressive tumor-immune microenvironment (TIME) - represent a crucial barrier to the effective treatment of post-operative glioblastoma, the most aggressive and lethal primary brain tumor in adults. The cavity left after tumor resection represents a valuable opportunity to deliver therapeutics locally via the placement of conformable scaffolds for the immediate chemotherapeutic targeting of GSCs and residual glioblastoma cells. We hypothesized that Surgiflo™, a moldable, FDA-approved gelatin-based hemostatic paste, could serve as a dual-purpose platform that minimizes postoperative bleeding and functions as a conformable, local, and sustained drug-delivery system. We incorporated a pH-sensitive designed hyaluronic acid-doxorubicin polymer-drug conjugate (HA-DOX) into the Surgiflo™ matrix, exploiting HA's selective affinity for CD44 (highly expressed by both GBM and GSCs) and promoting the depletion of GBM and GSCs via immunogenic cell death (ICD)-inducing properties of DOX. In vitro studies of HA-DOX-containing paste confirmed enhanced HA-DOX uptake in GSC-enriched models, improved ICD induction in GBM cells, favorable biocompatibility, and sustained drug release. In vivo evaluations revealed that intracavitary implantation of the HA-DOX-containing paste significantly prolonged the median survival of treated mice compared to the untreated group (37 days vs 25 days, respectively) and modulated the glioblastoma-associated TIME, resulting in a 44% reduction in GSC levels and a significant increase in CD8^+^ T cell levels (∗p < 0.5), compared to resected mice. Embedding HA-DOX within Surgiflo™ offers a promising strategy for localized, sustained delivery of chemotherapeutics to the resected cavity, with the potential to improve therapeutic outcomes and minimize systemic toxicity in postoperative glioblastoma treatment.

## Graphical abstract

**Figure:**
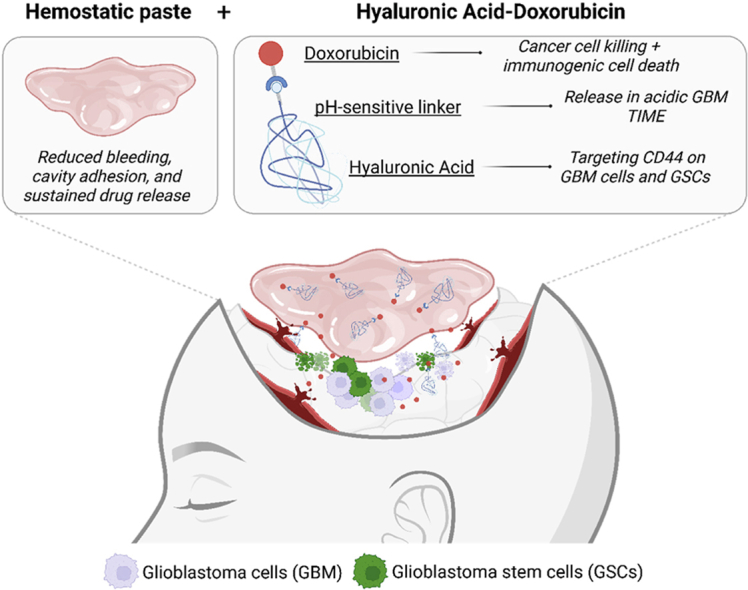

## 1 Introduction

The World Health Organization classifies glioblastoma (GBM) - the most aggressive and lethal primary brain tumor in adults - as a grade 4 glioma [1]. Despite the relative wealth of therapeutic approaches, including the conventional Stupp protocol (surgical resection, radiotherapy, and chemotherapy with temozolomide [2]) and more innovative strategies (e.g., anti-angiogenic agents like bevacizumab or tumor-treating fields [3,4]), GBM remains incurable, and disease recurrence remains inevitable [2]. Surgical resection represents a cornerstone of disease management, ranging from a minimally invasive biopsy to craniotomy with gross total resection [5]. Most recurrent tumors emerge at or near the resection margins, a region that remains hard to treat by current therapeutic strategies that aim to prevent/eliminate regrowth [6].

Many of the problematic characteristics of GBM derive from the tumor's highly infiltrative nature, marked intra- and inter-tumoral heterogeneity, and, most critically, the presence of glioblastoma stem cells (GSCs), a subpopulation of self-renewing, treatment-resistant cells that drive tumor propagation and tumor regrowth [7,8]. GSCs display inherent resistance to chemotherapy and radiotherapy through mechanisms including stem cell quiescence, enhanced DNA repair capacity, overexpression of drug-efflux transporters (e.g., ABC transporters), and protection by the hypoxic, immunosuppressive niche [9]. Studies have begun to detail the crosstalk between GSCs and components of the immunosuppressive tumor-immune microenvironment (TIME); for instance, GSCs polarize macrophages into the M2-like phenotype and secrete programmed death-ligand 1 (PD-L1)-containing extracellular vesicles, thereby contributing to local immunosuppression [10]. Meanwhile, the postoperative TIME promotes GSC turnover and stimulates angiogenesis, creating a pro-tumorigenic niche that accelerates disease recurrence [11].

Maximal safe resection – the standard treatment for GBM - offers an additional opportunity: postoperative local drug delivery into the resection cavity to target residual GBM cells and infiltrating GSCs. This strategy can bridge the gap in time between surgery and the start of chemotherapy/radiotherapy, as patients currently require at least three weeks for healing and immune stabilization [12]. The only FDA-approved treatment for local intracranial chemotherapy (Gliadel® wafers) displays limited survival benefits in recurrent GBM cases and lacks adaptability to the irregular geometry of post-surgical cavities [13]. Therefore, the application of conformable, moldable implants, such as pastes, presents a novel, promising alternative for intracavitary drug delivery in GBM patients [[14], [15], [16]].

**Fig. 1:**
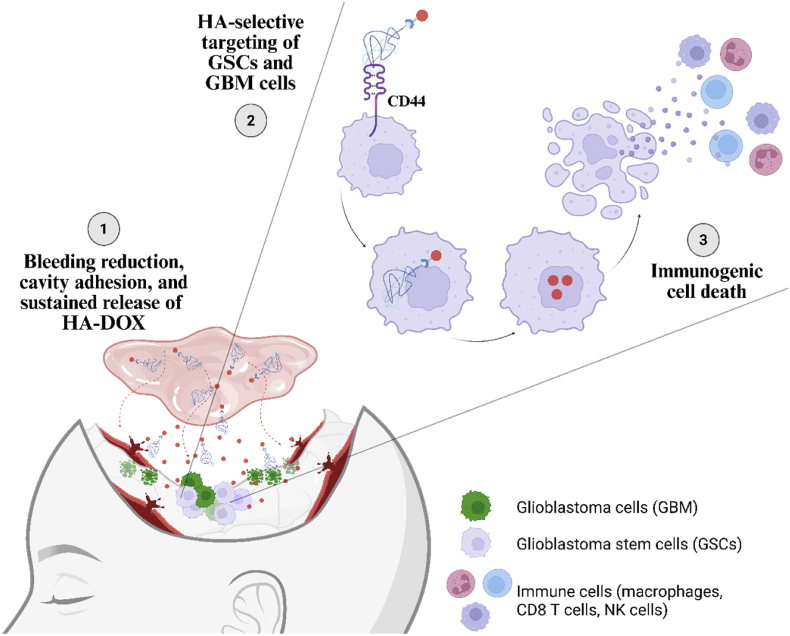
**Schematic representation of the paste-HA-DOX strategy**. A hemostatic paste embedded with HA-DOX locally reduces postoperative bleeding, induces sustained HA-DOX release to target GSCs, and promotes ICD.

Pastes, which typically comprise biodegradable polymers or biologically derived matrices [17], closely conform to the irregular surfaces of the resection cavity, facilitating intimate contact with residual tumor margins and potentially enhancing drug diffusion into infiltrative tumor zones. Here, we sought to apply Surgiflo™, an FDA-approved gelatin-based hemostatic paste containing thrombin used in neurosurgery, to the GBM resection cavity [[18], [19], [20], [21]]. Contact with blood fibrinogen initiates fibrin clot formation, a 3D polymerized network that reinforces the initial platelet plug and enhances hemostasis [18], through thrombin activity. Due to in-situ gel-forming abilities and adhesive properties, the fibrin matrix also presents an attractive platform for localized drug delivery [22]. Our strategy applies Surgiflo™ as a hemostatic agent to reduce bleeding in the cavity and function as a drug depot for the sustained, local release of therapeutics within the surgical site. Therapeutics may include free drugs or drug delivery systems, with the latter enabling sustained release within the cavity and protecting against premature drug leakage or wash-away [23,24]. In this context, we selected a polymer-drug conjugate that employs hyaluronic acid (HA), a naturally occurring biocompatible polysaccharide, as a polymeric carrier for the chemotherapeutic doxorubicin (DOX). HA enables selective targeting of DOX due to the high levels of CD44 expressed by GSCs [25,26] and GBM cells while DOX induces immunogenic cell death (ICD), which may add additional benefits by reprogramming the immunosuppressive TIME and promoting immune cell infiltration [27]. Previous studies have demonstrated the significant promise of locally treating GBM with HA-based drug conjugates, suggesting the potential for meaningful results [25,[27], [28], [29]]. Moreover, we hypothesize that GSC depletion will reshape the resected TIME; targeting immunosuppressive GSCs combined with DOX-induced ICD may enhance the infiltration of effector T cells, thereby contributing to improved treatment outcomes (Fig. 1).

Overall, our findings here indicate that integrating HA-DOX into Surgiflo™ produced a biocompatible paste that supports sustained drug release. When applied to the resected GBM cavity, this formulation prompted an increase in median survival in a mouse GBM model, which associated with TIME modulation and reduced GSC levels.

## 2 Materials and methods

### 2.1 Materials and reagents

HA (100 kDa) was purchased from Lifecore Biomedical, LLC (Chaska, MN, USA), and DOX was purchased from Chemielieva (Jiangbei, China). Paraformaldehyde (PFA) and crystal violet were purchased from Sigma-Aldrich (St. Louis, MO, USA). Ethylenediaminetetraacetic acid (EDTA), TRIzol™ reagent, Alamar blue, B-27™ Supplement (50X), N-2 Supplement (100x), human heat-stable fibroblast growth factor b (bFGF) recombinant protein, and cell culture media/reagents were purchased from Thermo Fisher Scientific (Waltham, MA, USA). Human recombinant epidermal growth factor (EGF) protein was purchased from Bio-Techne/R&D Systems (Dublin, Ireland). iFluor® 647-wheat germ agglutinin (WGA) conjugate was purchased from AAT Bioquest (Pleasanton, CA, USA). The Surgiflo™ Hemostatic Matrix was purchased from Ethicon (Raritan, New Jersey, USA). Tris borate EDTA (TBE), SybrSafe, and bovine serum albumin (BSA) were purchased from VWR International SRL (Leuven, Belgium). Endotoxin-free TE buffer was purchased from Qiagen (Hilden, Germany). The GoScript™ Reverse Transcription Mix, Oligo (dT) GoTaq® qPCR Master Mix, DeadEnd™ Fluorometric TUNEL System, and RealTime-Glo™ Extracellular ATP were purchased from Promega (Madison, WI, USA). μ-Slide Angiogenesis Uncoated slides were purchased from ibidi (Gräfelfing, Germany). XenoLight TM D-Luciferin potassium salt was purchased from Perkin Elmer (Shelton, CT, USA). Oligonucleotide primers were purchased from Integrated DNA Technologies (IDT) (Leuven, Belgium) and are listed in the Supporting Information (**SI**; Table S1). Table S2 lists the antibodies used and their providers**.**

### 2.2 Glioblastoma tumor cell and stem cell lines

*GBM Tumor Cells*. Green fluorescent protein (GFP)-expressing murine SB28 GBM cells (DSMZ, German Collection of Microorganisms and Cell Cultures GmbH, Leibniz Institute, Braunschweig, Germany) were cultured in Dulbecco's Modified Eagle Medium (DMEM) containing L-glutamine, 4.5 g/L D-glucose, and sodium pyruvate. The medium was supplemented with 10% fetal bovine serum (FBS), 100 U/mL penicillin, and 100 μg/mL streptomycin.

*GSCs*. GSCs were obtained as reported for other GBM cell lines [30]. Briefly, SB28 cells were cultured in serum-free DMEM medium supplemented with 1% 10,000 U/mL penicillin G and 10,000 μg/mL streptomycin, 1% B-27 supplement, 1% N-2 supplement, 0.1% 20 μg/mL of FGF, and 0.1% 20 μg/mL EGF (GSC-enriching medium). For GSC expansion, SB28 cells were seeded at a density of 500,000 cells/well in a 6-well plate. After 24 h, cells started to aggregate in neurospheres, and after 72 h, cells were collected and centrifuged at 300 g for 5 min at 4 °C. The supernatant was then removed, and the neurospheres were resuspended in GSC-enriching medium and seeded in a 12-well plate (500 μL/well). After 72 h, GSC neurospheres were ready for further investigations. SB28 cells and GSCs were incubated at 37 °C in a 10% CO_2_ atmosphere. To ensure the presence of GSCs, the expression of specific GSC marker genes (*Fut4* and *Klf4*) was evaluated by RT-qPCR (method described in the SI and results shown in Fig. S1).

### 2.3 In vivo orthotopic SB28 model and tumor resection procedure

All experiments were performed in accordance with the Belgian national guidelines and the EU Directive 2010/63/EU and approved by the ethical committee for animal care of the Faculty of Medicine of the Université Catholique de Louvain (2023/UCL/MD/65). Animals had free access to water and food and were monitored daily.

*Tumor inoculation procedure.* Immunocompetent C57BL/6J mice (female, six weeks old) purchased from Charles River Laboratories (Wilmington, MA, USA), were orthotopically grafted with 3 × 10^3^ SB28 cells/mouse (2 μL) into the right frontal lobe by convection enhanced delivery (CED) using a Hamilton syringe (26S gauge needle) mounted onto an infusion syringe pump (Harvard Apparatus, Holliston, MA, USA). Mice were anesthetized with an intraperitoneal injection of ketamine/xylazine (100 and 12 mg/kg) and positioned on a stereotactic frame. The injection was performed after drilling a hole into the skull with a surgical high-speed drill (Velleman, Gavere, Belgium) with the following coordinates: 2.1 mm lateral, 0.5 mm posterior from the bregma, and 2.6 mm deep from the outer border of the skull. On day 12, mice were randomized according to the tumor size into two groups (n = 9): untreated and resection.

*Tumor resection procedure.* Tumor resection was performed on day 14. All mice were anesthetized with an intraperitoneal injection of ketamine/xylazine (100 and 12 mg/kg, respectively) and a local subcutaneous injection of lidocaine (0.5%, 7 mg/kg) and fixed on the stereotactic frame. The skin was opened on a previous surgical scar, and the periosteum was removed to reveal the previous burr hole. A high-speed drill was used to gently break the skull around the burr hole, after which fine-tip tweezers (Dumont, Switzerland) were used to obtain a circular cranial window exposing the brain. A biopsy punch (2 mm Ø, Kai Medical, Germany) was inserted 2 mm deep and twisted to cut the brain/tumor tissue, and the explant was further removed with a vacuum pump (Vacuubrand GMBH + CO KG, Germany). Residual blood in the surgery cavity was removed using an absorbable hemostatic patch (Fine Science Tools, Germany). Afterwards, the dural window was repaired by covering it with a 4 × 4 mm square piece of Neuro-Patch® (Aesculap, Germany) impregnated with fibrin sealant (TISSEEL PRIMA; Baxter, France). The wound was then closed by suture. Mice were euthanized according to the endpoints of 20% body weight loss and clinical signs of morbidity (i.e., lack of movement, paralysis, and arched back). The remaining animals (n = 3) were sacrificed 3 days after tumor resection (day 17 from tumor inoculation) for histological and immunofluorescence analysis.

*Tumor imaging.* The presence and size of intracranial tumors were monitored by bioluminescence imaging using the IVIS Spectrum in vivo imaging system (Revvity, Waltham, MA, USA) and magnetic resonance imaging (MRI) with an 11.7 T Bruker Biospec MRI system (Bruker, Billerica, MA, USA). Details are reported in SI.

### 2.4 Histological and immunofluorescence analysis

At day 17, mice were sacrificed and brains were collected, fixed in 4% PFA (v/v in water), 10% sucrose, and 30% sucrose, and then finally embedded in OCT and stored at −80 °C. Brains were sectioned in 12 μm sections with a cryostat Cryostar NX70 (Thermo Fisher Scientific, Waltham, MA, USA) and collected on SuperFrost Plus glass slides. For histology, slides were stained with hematoxylin and eosin (H&E) (Fig. S2). For immunofluorescence, slides were treated with a blocking solution to avoid non-specific binding (10% goat serum, 10% BSA, and 0.2% Triton) for 1 h at room temperature. Then, staining with Alexa Fluor® 647 anti-human/mouse CD44 antibody, or Alexa Fluor® 647 anti-mouse CD133 antibody, or Alexa Fluor® 647 anti-mouse Nestin antibody, and DAPI was performed (Table S2). Slides were scanned using a Zeiss Axioscan 7 automated slide scanner (Oberkochen, Germany) and analyzed with QuPath software (Edinburgh, UK).

### 2.5 HA-DOX synthesis and characterization

HA-DOX was synthesized and characterized as previously described [[27], [28], [29]]. Table S3 provides information on the size, zeta potential, and drug-loading parameters of HA-DOX. All measurements were performed in triplicate. Yield: HA-DOX: 92 mg (%w/w DOX: 6.71%, HA: 93.29%).

### 2.6 Cytotoxicity studies

SB28 cells (5 × 10^3^ cells/well) were seeded in 96-well plates and incubated for 24 h. Cells were treated with DOX or HA-DOX in the 0.0001-10 μM range (DOX equivalents [equiv.]). After 72 h of treatment, cells were fixed with 4% PFA (v/v in water) and incubated with 50 μL/well crystal violet (0.5% w/v in 20% methanol v/v) for 30 min. Plates were then rinsed three times with ultrapure water and left to dry. Finally, crystals were solubilized in methanol, and the absorbance (*λ*_max_: 560 nm) was read with the SpectraMax ID5 microplate reader (Molecular Devices, San Jose, CA, USA). Assays were performed in three independent experiments, and the data were normalized to the untreated group (100% viability) and the Triton X-100 group (0% viability).

Similarly, GSCs were seeded (2 × 10^5^ cells/well) in a 96-well plate, and after 24 h, they were treated with DOX and HA-DOX in a 0.0001-50 μM range (DOX equiv.). After 72 h of treatment, the supernatant was removed, and alamarBlue™ Cell Viability Reagent (Thermo Fisher Scientific, US) was added to the cells, which were incubated at 37 °C for 24 h. Sample fluorescence was analyzed with a SpectraMax ID5 microplate reader (λ_ex_: 530 nm; λ_ex_: 590 nm).

### 2.7 Immunogenic cell death marker detection

*Calreticulin exposure.* SB28 cells (2 × 10^5^ cells/well) were seeded in 12-well plates and maintained in complete growth media for 24 h. Cells were then treated with 0.1 μM DOX and HA-DOX (DOX equiv.). After 24 h of treatment, flow cytometry (FACS) was employed to quantify the number of viable cells positive for calreticulin (CRT). Cells were detached from the plate using trypsin-EDTA, washed three times with PBS, and suspended in PBS supplemented with 5 mg/mL BSA and 100 μL 0.5 M EDTA (FACS buffer). Cells were stained with a primary anti-CRT antibody for 30 min at 4 °C, protected from light, and then with a secondary DyLight649-coupled antibody (DyLight™ 649 donkey anti-rabbit IgG, BioLegend, San Diego, CA, USA) for 30 min at 4 °C in the dark. Finally, data were acquired with a Cytek® Aurora flow cytometer (Cytek Biosciences, Fremont, CA) and analyzed with FlowJo software (FlowJo, Ashland, OR, USA) using a gating strategy to identify CRT-positive cells among viable GFP-expressing cells (Fig. S3).

*CRT detection* via *confocal microscopy.* SB28 cells (1 × 10^5^ cells/well) were seeded in a thin-bottom 96-well plate (Revvity, Waltham, MA, USA). Cells were then treated with 0.1 μM DOX and HA-DOX (DOX equiv.) or left untreated. After 24h, cells were washed with PBS, stained for their membrane using FITC-labelled Wheat Germ Agglutinin (WGA-FITC, 2 μg/mL, Life Technologies, Ref# W11261) for 5 min, washed again, fixed with 4% PFA, permeabilized using 0.2% Triton X-100 in PBS for 3 min, blocked using 5% BSA in PBS for 30 min, and stained with anti-CTR antibody-APC (Bio-Techne, Ref# NBP 1-47518APC) at a dilution of 1:100 in 1% BSA-PBS for 3 h. Finally, cells were washed and their nuclei marked with Hoechst 33342 (20 nM, Thermo Scientific, Ref# 62249) for 5 min. After the final wash, cells were imaged using a Zeiss LSM800 equipped with a 40x objective. For each condition, 8 images were acquired and processed in ImageJ by quantifying the median fluorescence intensity in the APC channel.

*ATP release.* SB28 cells (2 × 10^5^ cells/well) were seeded in 12-well plates and maintained in complete growth media. The following day, cells were treated with 0.1 μM DOX and HA-DOX (DOX equiv.). After 24 h, the supernatant was collected, and secreted extracellular ATP levels were measured with the RealTime-Glo™ Extracellular ATP Assay (Promega, Madison, WI, USA) using a Spectramax ID5 microplate reader, according to the manufacturer's instructions.

*Cxcl10 and Ifnβ expression.* SB28 cells seeded (2 × 10^5^ cells/well) in 12-well plates were treated with 0.1 μM DOX and HA-DOX (DOX equiv.). After 24 h of incubation at 37 °C, the measurement of *Cxcl10* and *Ifnβ* relative gene expression was performed via real-time quantitative polymerase chain reaction (RT-qPCR), as described in SI.

### 2.8 Evaluating DOX/HA-DOX uptake by GSCs

SB28 GSCs (2 × 10^5^ cells/well) were seeded in round-bottom plates with ultra-low attachment 96-well plates, centrifuged 1 min at 300 g to favor cell sedimentation, and incubated for 24 h with 5 μM DOX and HA-DOX (DOX equiv.) in GSC-enriched media. Following treatment, GSCs were stained with iFluor® 647-WGA conjugate (AAT Bioquest, US) to evaluate the presence of DOX and HA-DOX within cell membranes. For the competition study, a 3-h pre-treatment with 5 μM HA was performed before treatment with 5 μM DOX and HA-DOX (DOX equiv.) to saturate cell-surface CD44 receptors. Cells were then subjected to the same procedure to analyze the confocal microscope images. Intracellular colocalization of DOX/HA-DOX with GSCs markers (CD44 and Nestin) in SB28 GSCs was also investigated as reported in SI (Fig. S5). Images were analyzed with ZEN Blue software, and colocalization quantification was performed with ImageJ.

### 2.9 Paste preparation and studies

Surgiflo™ Hemostatic Matrix contains 4.0 g gelatin in a prefilled syringe and lyophilized thrombin for reconstitution in 2.0 mL saline solution (1000 UI/mL); together, these components yield a total of 8.0 mL paste. For the preparation of 500 μL paste (corresponding to 500 μg of paste), 350.0 mg gelatin, 178 μL thrombin (both Surgiflo™ Hemostatic Matrix), and 250 μL PBS were used (the blank paste comprised only gelatin and thrombin solution, without any DOX or PGA-DOX). Paste-DOX and paste-HA-DOX were prepared at a DOX dose of 5 μg/50 μL paste (considering 6% drug loading of DOX equiv. in HA-DOX). Thrombin and solutions of PBS/DOX/HA-DOX were solubilized together and then mixed with gelatin via connected Luer lock syringes to obtain the final formulation.

#### 2.9.1 Degradation study in cerebrospinal fluid-like medium by scanning electron microscopy

Blank paste and paste-HA-DOX were prepared as reported above. 100 μL paste was placed into a vial, and 10 μL fibrinogen solution (Tisseel, Fibrin Sealant, Baxter, USA) (90 mg/mL) was added to artificially trigger the fibrinolytic process. Pastes were then covered with 1 mL cerebrospinal fluid (CSF)-like medium at pH 5.5 or pH 7.4 and incubated at 37 °C (CSF-like medium composition has been previously described [31]). At pre-determined time points (0, 1, 4, and 7 days), pastes were removed from the medium and analyzed at 20 kV using a low vacuum (0.5-0.7 torr) by environmental scanning electron microscopy (ESEM – FEI Quanta 200, Hillsboro, Oregon, USA). Representative images (5 images per sample) were taken for each sample (n = 3) at every time point, and pore diameters were defined with ImageJ software.

#### 2.9.2 Drug release study in cerebrospinal fluid-like medium

After preparing each paste, DOX release was studied in CSF-like medium at pH 5.5 and 7.4 at 37 °C. At specific time points (0-720 h), 500 μL supernatant was collected and replaced with 500 μL fresh media. The samples were freeze-dried, dissolved in water, and stored at −20 °C for further use. Quantification was performed using high-performance liquid chromatography (HPLC) with a Shimadzu Prominence system (Shimadzu, Japan) equipped with a Nucleosil C18 column (Macherey-Nagel, Germany) (150 × 4.6 mm; particle size 5 μm). The mobile phase comprised 0.1% formic acid in acetonitrile (A) and 0.1% formic acid in water (B) with gradient elution (10% for B, 0 min; 90% for B, 13–15 min; 10% for B, 15–20 min). The flow rate was fixed at 0.6 mL/min, the detection wavelength was 480 nm, and the retention time was 3 min. The calibration curve was established with a limit of detection of 2.8 μg/mL, a limit of quantification of 8.4 μg/mL, and a correlation coefficient of *R*^2^ = 0.999.

#### 2.9.3 In vivo biocompatibility

Healthy mice were divided into two groups: resection only and resection + paste-HA-DOX (n = 3). Mice were monitored daily for 70 days and then euthanized to collect brains. Brains were fixed in 4% PFA (v/v in water), 10% sucrose, and 30% sucrose, then embedded in OCT and stored at −80 °C. Brains were sectioned in 12 μm sections with a cryostat Cryostar NX70 (Thermo Fisher Scientific, Waltham, MA, USA) and collected on SuperFrost Plus glass slides for further immunofluorescence staining. Slides were stained with DeadEnd™ fluorometric TUNEL assay (Promega, Madison, WI, USA) for cytotoxicity evaluation, anti-mouse Iba-1 rabbit primary antibody (Wako Pure Chemical Industries, Neuss, Germany) and anti-rabbit APC secondary antibody (Abcam, Cambridge, UK) for microglia, and anti-GFAP-Cy3 (Sigma Aldrich, St Louis, Missouri, USA) for astrocytes (Table S2). Slide analysis was performed with a Zeiss Axioscan 7 automated slide scanner (Zeiss, Oberkochen, Germany) and analyzed with Zen Blue software. Signal quantification was performed using QuPath software (Edinburgh, UK).

### 2.10 In vivo efficacy in the SB28-resected model

Immunocompetent C57BL/6J mice (female, 6 weeks old) purchased from Charles River Laboratories (Wilmington, MA, USA) were orthotopically grafted with 3 × 10^3^ SB28 cells/mouse (2 μL) into the right frontal lobe by CED, following the same procedure described above. On day 12, mice were randomized according to the tumor size into five groups (n = 7): untreated, resection, resection + blank paste, resection + paste-DOX, and resection + paste-HA-DOX. Tumor resection was performed on day 14 in all groups except the untreated group. Tumor surgery was performed as described above. For the resection group, no treatment was administered into the tumor cavity. For the other groups, 50 μg blank paste, 5 μg paste-DOX (DOX equiv.), or 5 μg paste-HA-DOX (DOX equiv.) were placed into the resected cavity. Afterwards, the wound has been closed following the same procedure described in paragraph 2.2. Tumor growth was monitored over time by IVIS, and the survival rate was assessed. On day 26 after tumor inoculation, an MRI was performed to assess the presence of pastes in the cavity.

### 2.11 In vivo tumor-immune microenvironment study in the SB28-resected model

After establishing the SB28 orthotopic model as described above, mice were randomized into five groups (n = 8): untreated, resection, resection + blank paste, resection + paste-DOX, and resection + paste-HA-DOX. On day 17, mice were euthanized, and brains were collected to assess the effects of the treatments on GSCs and immune cells by FACS.

*Flow cytometry.* Single-cell suspensions of collected brains (n = 4/5) were obtained via mechanical dissociation and passage through a 70-μm cell strainer (Greiner Bio-One, Vilvoord, Belgium). Only the tissue around the tumor cavity was considered. Cells were stained with a panel of antibodies (Table S2) for 30 min at 4 °C to quantify distinct cell populations. Stained cells were then fixed with 4% PFA (v/v in water), and samples were analyzed at Cytek® Aurora flow cytometer (Cytek Biosciences, Fremont, CA) by acquiring 100,000 events/sample and analyzed with FlowJo software (FlowJo, Ashland, OR, USA). Figs. S6 and S7 describes the gating strategies employed.

### 2.12 Statistical analysis

Statistical analyses were performed using GraphPad Prism (GraphPad Software Version 9.1.2, San Diego, CA, USA). Survival was plotted using Kaplan–Meier curves and analyzed with a log-rank (Mantel-Cox) test. Multiple comparisons were assessed using a one-way ANOVA with Dunnett's multiple-comparison test. Statistical significance was indicated as follows: ∗p < 0.05, ∗∗p < 0.01, ∗∗∗p < 0.001, ∗∗∗∗p < 0.0001.

## 3 Results

### 3.1 The SB28-based glioblastoma resection model possesses GSC-like features

**Fig. 2:**
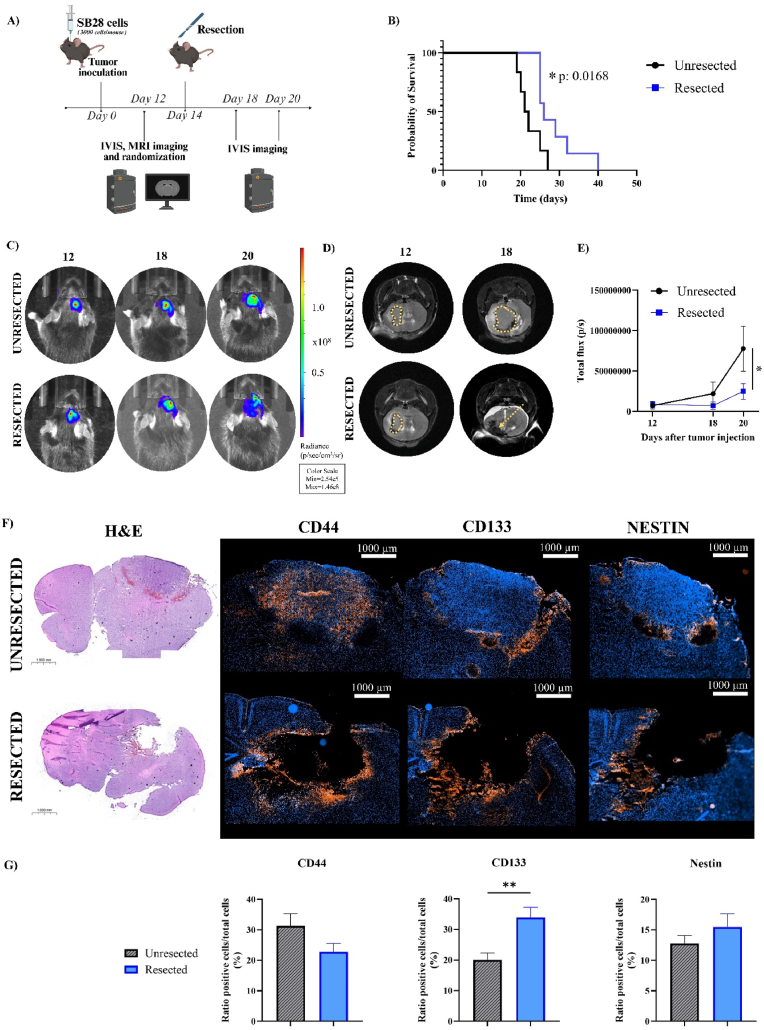
**The SB28-based GBM resection model provides evidence of increased mouse survival and GSC presence. A**) Schematic representation of the protocol followed for SB28 cell inoculation, tumor growth, and tumor resection on day 14 and IVIS and MRI imaging over time. **B**) Kaplan-Meier survival curves for unresected (n = 6) and resected (n = 6) mice. Statistical analyses performed using a log-rank (Mantel-Cox) test (∗P < 0.05). **C**) Representative images of tumor growth obtained by IVIS imaging on days 12, 18, and 20. **D**) Representative images of tumor growth obtained by MRI on days 12 and 18. **E**) Tumor growth curves for unresected and resected mice analyzed via total flux measured by IVIS on days 12, 18, and 20. Statistical analyses performed with Two-way ANOVA and Sidak's multiple comparisons tests (∗P < 0.05). **F**) Representative immunohistochemistry and immunofluorescence images for unresected and resected tumors with H&E, CD44, CD133, and Nestin staining. **G)** Graphs representing the % ratio of positive cells to CD44, CD133, or Nestin relative to the total amount of cells for the unresected and resected group, analyzed with QuPath software. Statistical analyses performed with an unpaired *t*-test (∗∗p < 0.01).

We developed a GBM resection model employing tumors derived from SB28 cells, which express GFP and luciferase and exhibit a heightened immunosuppressive phenotype, thereby providing a more accurate recapitulation of the in vivo tumor immune microenvironment (TIME) than other commonly used GBM cell lines (e.g., GL261) [32]. Importantly, SB28 cells exhibit low immunogenicity and display resistance to immune checkpoint inhibitors [33]. We orthotopically grafted mice with SB28 cells and performed tumor resection on day 14 after tumor inoculation (Fig. 2A)**.** Tumor resection provided relief from the tumor burden, resulting in a significant increase in median survival (∗p < 0.05) for resected (26 days) compared to unresected (21.5 days) mice (Fig. 2B).

We monitored tumor progression over time via tumor cell luciferase expression, which showed lower signal intensities in the resected group compared to the unresected group, where tumors grew more rapidly (Fig. 2C). Although we removed the initial tumor mass in resected mice, residual and infiltrating tumor cells persist and support tumor regrowth. The unresected group exhibited rapid and continuous tumor progression, whereas tumor regrowth occurred more slowly following surgical removal in the resected group. On day 18, MRI images revealed the resected cavity as a darker region, whereas the unresected group showed a clear growing tumor mass (Fig. 2D). Notably, the recurrent tumors in the resected group remained significantly smaller than those in the unresected group on day 20 (∗p < 0.05) (Fig. 2E).

We performed immunohistochemical analysis on brain sections from both unresected and resected SB28 tumor-bearing mice (Fig. 2F). We inoculated tumors and performed surgical resection in the right hemisphere, so while we applied staining to the entire brain, we selected only those regions with unresected tumors or resection cavities as representative images. We employed H&E staining to assess histological architecture and immunofluorescence to detect cells expressing glioblastoma stem cell (GSC) markers (CD44, CD133, and Nestin). Increased cellularity (as evidenced by dense hematoxylin staining) indicates the presence of tumor tissue in unresected mice; however, we did not observe this feature in resected mice (Fig. 2F). We qualitatively and quantitatively assessed the number of cells expressing those GSC markers through image analysis (Fig. 2G). Overall, mice with unresected and resected tumors displayed a similar amount of CD44^+^ cells. While CD133^+^ cells were predominantly located near the tumor margins in unresected mice, we observed a significant increase (∗∗p < 0.01) in the number of CD133^+^ cells in the tumor cavity after resection, suggesting a role for resection in triggering an increase in the number of tumor-initiating GSCs. We observed a lower number of Nestin + cells (compared to CD44^+^ and CD133^+^ cells) in unresected mice, and this number remained similar in the resected cavity. Overall, this newly established SB28-based GBM resection model provides the rationale for targeting GSCs within the surgical margins.

### 3.2 HA-DOX treatment induces immunogenic cell death and depletes GSCs

**Fig. 3:**
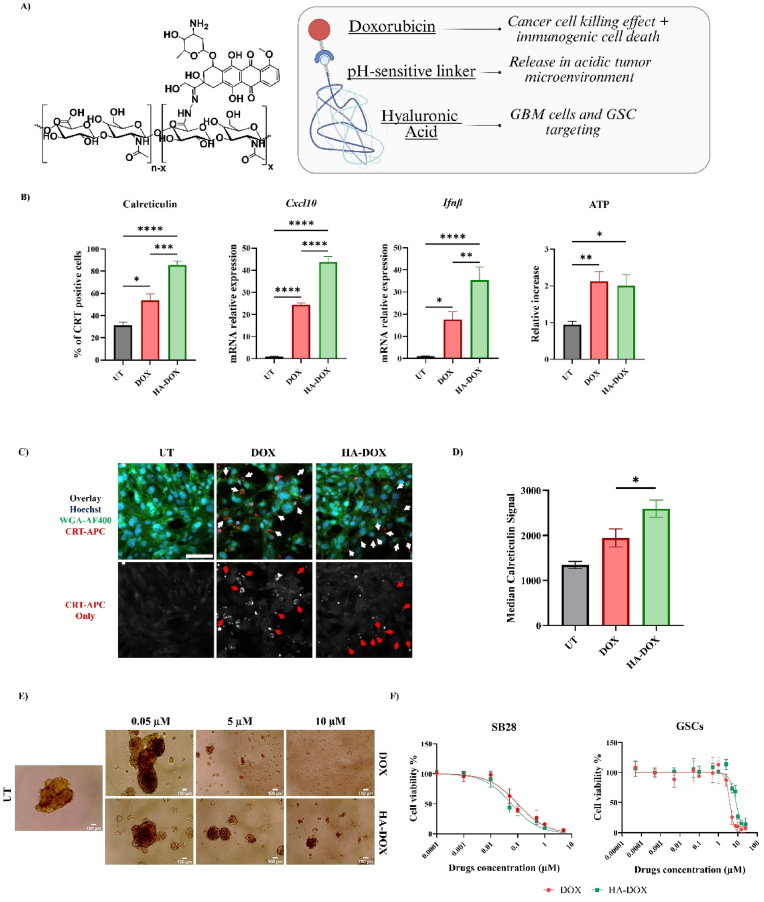
**HA-DOX induces immunogenic cell death in SB28 cells and cytotoxicity in GSCs. A**) Chemical structure of the HA-DOX conjugate and a graphical representation of the therapeutic rationale. **B**) Calreticulin (CRT) exposure expressed as percentage of positive cells among live cells, *Cxcl10* and *Ifnβ* gene expression, and ATP relative release after treatment of SB28 cells with 0.1 μM DOX or HA-DOX. Values (n = 3) represented as mean ± SD. **C)** Representative confocal images of SB28 cells after treatment with DOX or HA-DOX at 0.1 μM (DOX equiv.) for 24 h. Cells were stained for CRT (in red); cell membranes were marked with WGA-AF488 (in green); and nuclei were labelled with Hoechst (in blue). 40X magnification. Scale bar = 100 μm. **D)** Median CRT fluorescence intensity quantified using ImageJ (n = 3). Statistical analyses performed with an unpaired *t*-test (∗p < 0.05). **E**) Representative images of untreated SB28-derived GSCs (as neurospheres) treated with DOX or HA-DOX at 0.05, 5, and 10 μM (DOX equiv.), acquired with an electronic microscope (10X magnification). **F**) Cell viability curves after 72 h of incubation of SB28 bulk tumor cells or SB28-derived GSCs with DOX or HA-DOX at a concentration range of 0.0001-100 μM (DOX equiv.). Values (n = 6 for SB28 cells; n = 3 for GSCs) represented as mean ± SD. All statistical analyses performed with one-way ANOVA with Dunnett's multiple comparison tests (∗p < 0.05, ∗∗p < 0.01, ∗∗∗p < 0.001, ∗∗∗∗p < 0.0001).

We designed the HA-DOX conjugate to target the CD44 receptor expressed by GBM bulk tumor cells and GSCs [25,34], thereby allowing DOX to induce immunogenic cell death (ICD) [27]. We conjugated hyaluronic acid (HA) and doxorubicin (DOX) via a pH-sensitive linker to achieve selective drug release in the acidic GBM tumor microenvironment [28] (Fig. 3A). The drug loading has been determined by UV-VIS. We were able to conjugate 5.99% w/w DOX loading to HA. Superior drug loading led to a decrease in the water solubility of the conjugate, reducing its applicability. The HA-DOX conjugate exhibited an average diameter of 15.9 nm and a zeta potential of −21.4 mV. The relatively small size of HA-DOX is likely attributable to the hydrophobicity of DOX (Log P = 1.3) that influences the molecular conformation of the conjugate in solution [35,36]. Notably, this nanoscale size may enhance penetration into the tumor brain parenchyma, particularly given that microglial cells exploit CD44 signaling for proliferation [37]. Furthermore, the negative zeta potential suggests that HA-DOX uptake is predominantly mediated through receptor-dependent mechanisms. To demonstrate the ICD-inducing properties of HA-DOX, we analyzed ICD markers (calreticulin [CRT] exposure, *Cxcl10* and *Ifnβ* mRNA expression, and extracellular ATP release) in SB28 cells (Fig. 3B). We evaluated the ICD effect of HA-DOX in SB28 GBM cells rather than GSCs, as this was considered more biologically relevant. Although GSCs are key drivers of recurrence, they constitute only a small fraction of GBM in patients (approximately 0.3–1.8% CD133^+^ cells). Consequently, we hypothesized that the ability of HA-DOX to activate innate immune responses is likely driven primarily by its effects on GBM cells rather than on GSCs [38]. HA-DOX treatment (0.1 μM DOX equiv.) significantly increased CRT exposure compared to the untreated (∗∗∗p < 0.0001) and DOX-treated (∗∗∗p < 0.001) groups; DOX also significantly increased CRT exposure compared to the untreated group, but to a lesser extent (∗p < 0.05).

Comparable behavior was also confirmed by confocal microscopy analysis: HA-DOX induced a significantly higher signal of CRT exposure (∗p < 0.05) compared to the DOX-treated group (Fig. 3C and D, and S4). HA-DOX treatment induced higher *Cxcl10* (∗∗∗∗p < 0.0001 vs. untreated and DOX groups) and *Ifnβ* (∗∗∗∗p < 0.0001 vs. untreated; ∗∗p < 0.01 vs. DOX group) expression, further highlighting the enhanced immunostimulatory potential relative to both control and DOX-treated conditions. Of note, DOX significantly increased *Cxcl10* (∗∗∗∗p < 0.0001) and *Ifnβ* (∗p < 0.05) expression compared to the untreated group. These findings suggest that HA can enhance DOX-mediated ICD, likely through improved cellular uptake. In contrast, we found comparable levels of extracellular ATP release between the DOX and HA-DOX groups, although levels remained significantly higher than those in untreated cells (∗∗p < 0.01 and ∗p < 0.05), indicating that HA conjugation did not compromise the effect.

Next, we developed a method to expand SB28-derived GSCs in vitro as neurospheres using a specific cancer stem cell-enriching medium [[29], [30], [31]]; this approach supported the formation of neurospheres with a 3D structure and a spherical/ovoid shape. The study of cytotoxicity in expanded SB28-derived GSCs revealed a comparable effect of DOX (IC_50_: 3.881 ± 0.235) and HA-DOX (IC_50_:8.156 ± 0.546) treatment, as shown by the representative images with increasing concentrations (Fig. 3E); however, SB28-derived GSCs exhibited reduced sensitivity compared to parental SB28 cells (DOX IC_50_: 0.09995 ± 0.0163; HA-DOX IC_50_: 0.05876 ± 0.0089), with a higher concentration threshold required to elicit a therapeutic response and a higher IC_50_ for HA-DOX (Fig. 3F)**.**

Overall, the findings demonstrate that HA-DOX preserves, and may even enhance, the ICD-inducing effects of DOX in SB28 cells, as highlighted by our analyses of CRT exposure, *Cxcl10* and *Ifnβ* expression, and ATP release. Additionally, HA-DOX exhibits cytotoxicity against SB28-derived GSCs, although requiring higher concentrations than for parental SB28 cells.

### 3.3 HA-DOX selectively targets GSCs by interacting with CD44

**Fig. 4:**
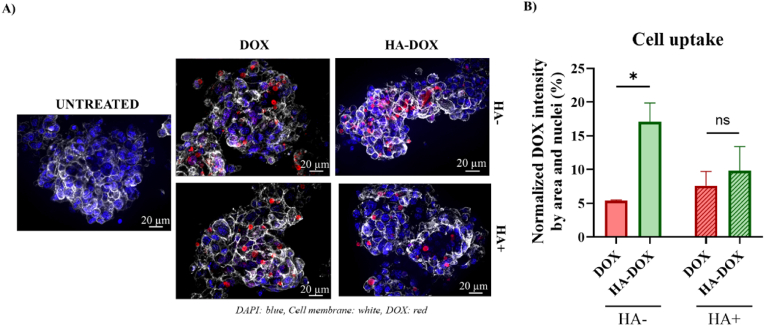
**Evaluation of HA-DOX internalization by in neurospheres enriched in GSCs. A**) Representative images of DOX/HA-DOX uptake in GSCs with/without HA pre-treatment for 3 h. DOX staining in red; cell membranes stained in white; Nuclei stained with DAPI (blue). 40X magnification; Scale bar = 20 μm. **B**) Normalized DOX intensity by analyzed area and nuclei when pretreating GSCs with/without HA (n = 3). Statistical analyses performed with an unpaired *t*-test (∗p < 0.05; ns – not significant).

We next aimed to understand the mechanism of action of HA-DOX by performing confocal microscopy-based analyses to evaluate the targeting ability of DOX and HA-DOX toward glioblastoma stem cells (GSCs) in vitro. Although CD44 is also in microglia and astrocytes, its levels are substantially lower than in GBM and stem cells; therefore, HA is expected to preferentially deliver DOX to tumor cells [39]. Moreover, any limited off-target uptake is unlikely to cause significant toxicity, as DOX primarily affects rapidly dividing cells, and healthy cells exhibit low proliferative activity compared to highly proliferative SB28 cells. We observed intracellular colocalization of DOX with CD44 or Nestin, indicating the specific internalization of the drug by cells expressing GSC markers (Fig. S5). We further evaluated cell uptake by staining cell membranes in the presence or absence of HA pre-treatment to explore the relevance of HA-DOX targeting of CD44 (Fig. 4A and B). In the absence of HA pre-treatment, HA-DOX demonstrated significantly higher internalization relative to DOX (Fig. 4A), as evidenced by normalized intracellular fluorescence intensity (∗p < 0.05) (Fig. 4B); however, pre-treatment with HA, which blocks CD44 interactions, reduced uptake, suggesting that HA-DOX:CD44 interactions may play a significant role in internalization, even though other pathways likely play some role. Overall, these findings highlight the receptor-specific mechanism underpinning the preferential uptake of HA-DOX in GSCs.

### 3.4 A biocompatible and degradable paste scaffold supports sustained DOX release

We selected Surgiflo™, an FDA-approved paste scaffold, to fill the resection cavity. Compared to other locoregional treatments for GBM like Gliadel® wafers, this approach has the advantage of better adherence to the brain walls, while exerting a hemostatic effect. Moreover, it serves as a localized depot system for direct DOX/HA-DOX delivery, ensuring a more uniform drug distribution at the tumor margins where recurrence is most likely to occur. Moreover, the presence of HA-DOX enhances the targeting properties towards GBM and GSC cells while eliciting an ICD effect [20,40,41].

**Fig. 5:**
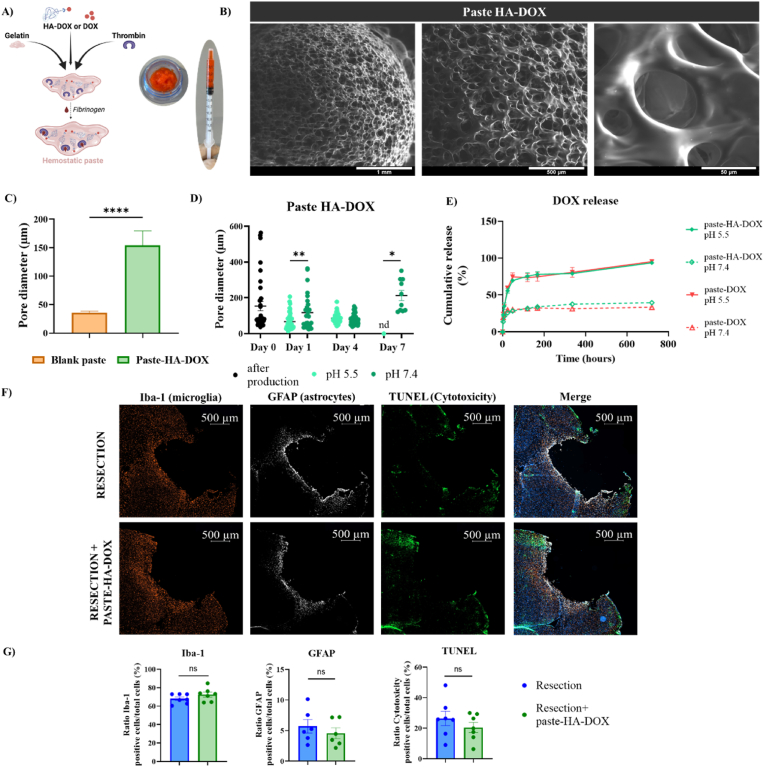
**Biocompatible and degradable hemostatic paste scaffold induces sustained cargo release. A**) Schematic representation of paste preparation and representative images of the hemostatic paste. **B**) Representative SEM images of paste-HA-DOX. **C**) Pore diameter (expressed in μm) of blank paste and paste-HA-DOX after production, analyzed from representative SEM images (5 images per sample) captured for each sample (n = 3) via ImageJ analysis of pore diameter. Statistical analyses performed via an unpaired *t*-test (∗p < 0.0001). **D**) Paste-HA-DOX degradation over time in CSF-like medium at pH 5.5 or 7.4 expressed in terms of pore diameters. Representative SEM images (5 images per sample) captured for each sample (n = 3) at each time point. Statistical analyses performed via an unpaired *t*-test at each time point at pH 5.5 and 7.4 (∗p < 0.05, ∗∗p < 0.01). **E)** Cumulative cargo release from paste-DOX and paste HA-DOX over time in vitro following incubation in CSF-like medium at pH 5.5 or 7.4. **F**) Representative brain cavity images depicting in vivo biocompatibility in healthy mouse brains following resection and resection and treatment with paste-HA-DOX according to Iba-1 (microglia; orange), GFAP (astrocytes; white), and TUNEL (cytotoxicity; green) signals. **G)** Graphs representing the % ratio of positive cells to Iba-1, GFAP, and TUNEL, relative to the total number of cells. Statistical analyses performed with an unpaired *t*-test (ns – not significant).

We prepared each paste by mixing the gelatin and thrombin solution with PBS (paste blank), DOX (paste-DOX), or HA-DOX (paste-HA-DOX). Upon contact with blood, fibrinogen (or fibrinogen added to the formulation) formed a fibrin matrix, yielding the final hemostatic scaffolds (Fig. 5A). We studied the morphology of each paste by scanning electron microscopy (SEM), revealing a highly porous and interconnected structure that appeared relatively homogenous throughout the scaffold bulk (Fig. 5B depicts representative SEM images of paste-HA-DOX). Regarding morphology, paste-HA-DOX exhibited significantly larger pore diameters than the blank paste (HA-DOX: 154.12 μm vs. blank: 35.79 μm; ∗∗∗∗p < 0.0001), likely due to the hydrophilic nature of HA, which promotes water interaction and porous network formation (Fig. 5C).

We performed degradation studies for paste-HA-DOX in cerebrospinal fluid (CSF)-like conditions to simulate the post-surgical environment of the resected brain cavity; to reflect physiological variation, we employed a more acidic pH (5.5) to simulate the enhanced acidity typically found in the inflammatory phase that occurs after the surgery in the resected cavity and a neutral pH (7.4) to represent the conditions of healthy brain tissue (Fig. 5D) [42]. We monitored changes in pore diameter as an indicator of degradation over time, since pore enlargement or disappearance reflects structural destabilization caused by water dissolution or matrix erosion (Fig. 5D). At day 1, paste-HA-DOX samples incubated at pH 7.4 exhibited significantly larger pores (120.8 μm) compared to those at pH 5.5 (63.8 μm; ∗∗p < 0.01). By day 4, pore sizes at both pH conditions compared well (pH 7.4: 67.6 μm; pH 5.5: 74.3 μm), indicating a similar degree of structural integrity; however, by day 7, while paste-HA-DOX at pH 5.5 underwent complete degradation (pores no longer detectable), the formulation at pH 7.4 persisted with markedly enlarged pores (221.9 μm; ∗p < 0.05), reflecting ongoing but incomplete degradation.

We then compared free DOX release from paste-DOX and paste-HA-DOX in CSF-like medium at pH 5.5 and 7.4 (Fig. 5E). Paste-DOX and paste-HA-DOX revealed burst release over the first 24 h at pH 5.5, reaching 58.9% (DOX) and 55.7% (HA-DOX) release, followed by sustained, gradual release over the next 720 h (corresponding to one month). In contrast, we observed a lower level of burst release of 29.7% (DOX) and 25.7% (HA-DOX) over the first 24 h at pH 7.4, with no significant increase observed over the subsequent experimental timeframe (till 720 h) (Fig. 5E–S8).

Since the standard in vitro rheological characterization is difficult due to the fibrin clot formation (triggered by the external addition of fibrinogen), which induces a rapid transition from a paste-like state to a semi-solid clot, we decided to assess the biocompatibility of the HA-DOX–containing paste through an in vivo study assessing the presence of secondary injury. Subsequent biocompatibility assessments in healthy resected brain tissue revealed comparable outcomes between resected mice and those not receiving treatment, and those treated with paste-HA-DOX. Fig. 5F provides representative immunostaining images, while Fig. 5 G describes the quantification of these findings. Overall, analysis of markers of neuroinflammation and cytotoxicity - Iba-1 for microglial activation, GFAP for astrocyte activation, and the TUNEL assay (which detects DNA fragments after cell death) - failed to reveal significant differences between the two groups, supporting the biocompatibility of paste-HA-DOX.

Overall, paste-HA-DOX exhibited greater porosity than the blank paste, likely due to the hydrophilic nature of HA, which enhances water absorption and pore size. Under acidic conditions, paste-HA-DOX exhibited a faster degradation profile than at neutral pH, primarily boosting and then sustaining DOX release. In contrast, the higher structural stability at neutral pH, combined with electrostatic interactions between gelatin (which has an isoelectric point between 8 and 9, suggesting a net negative charge at physiological pH) and DOX (which has a pKa around 9.93, suggesting a net positive charge at physiological pH), led to slower drug release from paste-HA-DOX [43,44]. Moreover, biocompatibility in healthy resected brain supports the potential application of paste-HA-DOX as a scaffold for the delivery/release of therapeutics.

### 3.5 Paste-HA-DOX treatment increases median survival in the SB28-based GBM resection model

**Fig. 6:**
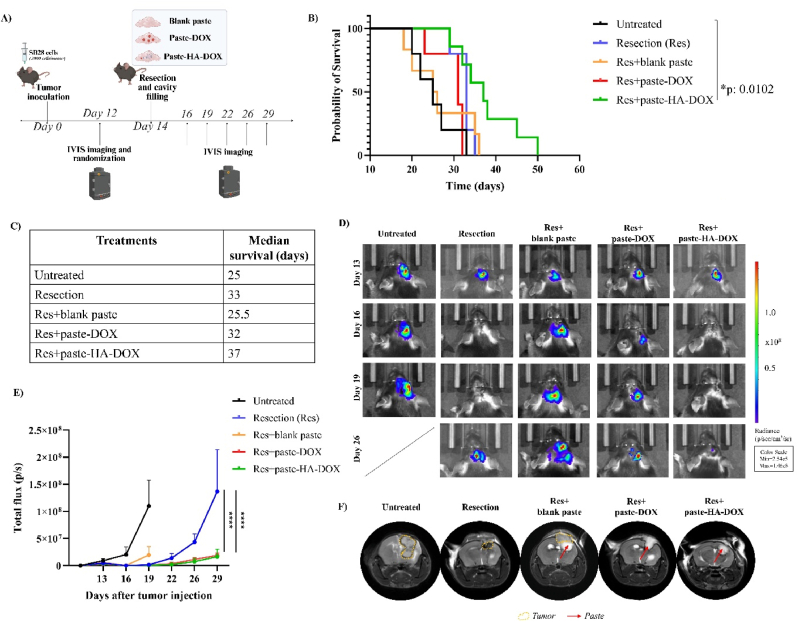
**Paste-HA-DOX significantly extended the survival in SB28-resected mice. A**) Schematic representation of the in vivo protocol followed in this experiment. **B**) Kaplan-Meier survival curves for untreated (n = 5), resected (Res) (n = 5), Res + blank paste (n = 6), Res + paste-DOX (n = 5), and Res + paste-HA-DOX (n = 7) mice. Data analyzed with a log-rank (Mantel-Cox test). **C)** Table describing median survival values (expressed in days). **D**) Representative IVIS images of the brains at day 13, 16, 19, and 26 after tumor inoculation. **E)** Analysis of tumor growth via total flux measured by the IVIS system on days 13, 16, 19, 22, 26, 29 after tumor inoculation (tumor resection and cavity filling performed on day 14). **F)** Representative MRI images of the brains on day 26, with tumors circled in yellow and paste presence indicated via a red arrow. Statistics performed with Two-way ANOVA and Tukey's multiple comparisons (∗p < 0.05; ∗∗∗∗p < 0.0001).

We next explored the efficacy of paste-DOX and paste-HA-DOX treatment in our SB28-based GBM resection model. We orthotopically grafted mice with SB28 cells and resected them on day 14; we then treated them with blank paste, paste-DOX, or paste-HA-DOX (Fig. 6A). In addition, resected mice did not receive any treatment, while untreated mice did not undergo resection. We monitored animal survival and tumor growth over time using IVIS imaging and MRI. No differences in body weight were observed in any of the groups (Fig. S9). The resected animals treated with paste-HA-DOX (Res + paste-HA-DOX) demonstrated a significant increase in survival (∗p < 0.05, median survival: 37 days) compared to the untreated group (median survival: 25 days), although other groups displayed no significant alterations (Fig. 6B and C)**.** We observed comparable survival between untreated and blank-paste-treated mice, which may reflect the 'cancer cell trap' effect, in which an empty matrix promotes cancer cell proliferation [[45], [46], [47]]. We observed enhanced control over tumor growth in resected mice treated with paste-DOX (Res + paste-DOX) and paste-HA-DOX (Res + paste-HA-DOX) compared to resected (Res) animals at day 29 (both ∗∗∗∗p < 0.0001) (Fig. 6D and E, individual mice tumor growth curve is represented in Fig. S10). It is noteworthy that, although no statistically significant difference in tumor volume is observed between the Resection + paste-DOX and Resection + paste-HA-DOX groups at day 29, a separation of their survival curves is evident. This pattern suggests that both treatments elicit a comparable initial response to local chemotherapy in the early post-operative period, whereas differences in their therapeutic effects may emerge at later time points. Representative MRI images demonstrated more effective tumor growth control in the Res + paste-HA-DOX and Res + paste-DOX groups, with the paste clearly visible in the resection cavity (indicated by a red arrow in Fig. 6F). In contrast, analysis of the Res + blank paste group revealed paste retention accompanied by evident tumor recurrence surrounding the cavity (circled by a yellow line in Fig. 6F).

Overall, the tumor volumes between the Resection + paste-DOX and Resection + paste-HA-DOX groups do not show a statistically significant difference. This is consistent with our survival data, as the initial response to local chemotherapy is comparable in both groups during the early post-operative phase. However, a clear divergence in the therapeutic profiles becomes apparent at later time points.

In summary, filling the resection cavity with paste-HA-DOX led to a notably improved median survival and more effective tumor growth control compared with untreated animals. These findings offer encouragement for the treatment of resected GBM and support the hypothesis that Surgiflo™ serves as a depot for sustained drug delivery in the GBM resection cavity.

### 3.6 Paste-HA-DOX modulates the tumor immune microenvironment and GSC number

**Fig. 7:**
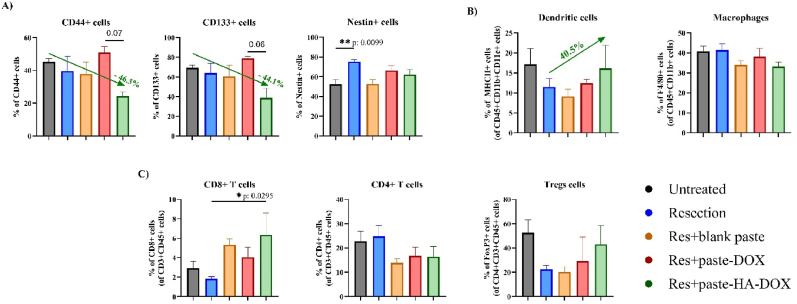
**GSC and immune cell modulation in the treated GBM microenvironment.** FACS analysis of **A**) GSCs via CD44/CD133/Nestin expression, **B**) myeloid cells (dendritic cells and macrophages), and **C**) lymphoid cells (CD8^+^ T cells, CD4^+^ T cells, and Treg cells) in the GBM-associated TIME at day 17, expressed as percentage of positive cells. Values (n = 5) represented as mean ± standard error of the mean (SEM). Statistical analyses performed using one-way ANOVA with Dunnett's multiple comparison test (∗p < 0.05; ∗∗p < 0.01).

Finally, we investigated the mechanism underlying the anti-tumor efficacy of paste-HA-DOX by assessing the tumor immune microenvironment (TIME) of the resection cavity 3 days after tumor resection and treatment. Flow cytometry analysis of brain tissues revealed that treatment with paste-HA-DOX reduced the number of presumptive CD44^+^ GSCs in the cavity compared to the untreated control (46.3% reduction) (Fig. 7A). Although we did not observe a significant difference, the reduction compared with the paste-DOX treatment approached significance (p = 0.07), suggesting an improvement following HA conjugation. CD133 expression levels displayed no significant differences across groups, although we observed a reduction in the number of CD133^+^ cells following paste-HA-DOX treatment compared to the untreated control (44.1% reduction for paste-HA-DOX) and approaching a significant reduction compared to paste-DOX treatment (p = 0.06). For Nestin expression, we observed similar levels in untreated and paste-HA-DOX-treated groups but a significant increase after resection (∗∗p < 0.01) (Fig. 7A). When studying the myeloid population of antigen-presenting cells, we did not observe significant differences across groups; however, we did observe a trend toward increased numbers of dendritic cells following paste-HA-DOX treatment compared with resected mice; furthermore, the macrophage cell population remained unchanged (Fig. 7B). In lymphoid cells, we observed a statistically significant increase in CD8^+^ T cell numbers following paste-HA-DOX treatment compared to resected mice, but no alterations in CD4^+^ T cell or T regulatory cell (Treg) numbers (Fig. 7C). Although we failed to detect significant perturbations of the TIME regarding GSCs or immune cell populations, treatment of the resected cavity with paste-HA-DOX revealed encouraging trends suggesting a potential reduction in GSCs and a concomitant increase in immune cells. These preliminary observations warrant further investigation to validate and better understand the underlying mechanisms.

## 4 Discussion

The placement of implantable depots into the resected GBM tumor cavity allows for prolonged drug retention, improved efficacy, and reduced systemic toxicity, thereby minimizing disease recurrence. Incorporating polymer-drug conjugates, such as HA-DOX, into these depots enables further control over drug release kinetics and introduces functionalities such as cell-specific targeting and immunogenic cell death (ICD) induction [48]. In our study, we present a novel strategy that employs a hemostatic paste scaffold, Surgiflo™, embedded with a hyaluronic acid – doxorubicin (HA-DOX) conjugate that exhibits dual, synergistic functionality, aiming to overcome the therapeutic gap between surgery and the initiation of adjuvant treatment in GBM. Previously, other hemostatic biomaterials have been used to fill the GBM resection cavity [49]. The innovation of our approach lies in 1) combining local cavity filling and hemostatic action, embedding a polymer drug conjugate, providing sustained drug release, and 2) strong clinical translatability, as achieving haemostasis during brain surgery is essential to prevent postoperative re-bleeding and poor outcomes [50]. Notably, the use of HA enables GSC and GBM bulk tumor cell targeting via interactions with CD44, thereby allowing the eradication of residual, invasive tumor-initiating cells that contribute to recurrence. DOX also promotes anti-tumor efficacy by inducing ICD, potentially recruiting innate immune responses to the tumor site, and enhancing tumor cell clearance. Despite DOX's beneficial properties as a robust anticancer drug, several preclinical and clinical studies have reported that DOX remains largely ineffective at eliminating cancer stem cells (CSCs) [34,35], and the clinical application of DOX often leads to the emergence of drug-resistant GSCs [51]. Here, the rationale for conjugating DOX to HA to generate a new "macromolecular prodrug" overcoming resistance in this cell subset while promoting a local immune response.

Here, we used a newly developed orthotopic GBM resection model based on the formation of SB28 cell-derived tumors, which exhibit low immunogenicity and a limited mutational burden, making them suitable for modeling the immune landscape and the treatment resistance observed in human GBM, especially in the context of immunotherapy [32]. While the GL261 model is highly immunogenic and responsive to various immunotherapies, the SB28 model reflects the low mutational burden, poor T-cell infiltration, and inherent resistance to immune checkpoint inhibitors seen in the majority of GBM patients [32,52]. Performing surgical resection 14 days post-tumor inoculation induced a significant increase in median survival, but not a curative effect. Histological analysis of the unresected tumor has revealed classical features of human GBM, including necrosis, pleomorphism, hyperproliferation, and invasiveness [53]. While various GSC-like models have been evaluated [54], our study explores a GBM tumor model that preserves immunosuppressive features while having high rates of GSCs, driving force of GBM recurrences [55]. Notably, we detected expression of GSC-associated markers (CD44, CD133, and Nestin) throughout disease progression, providing a rationale for studying the efficacy of our strategy in this SB28-resected model.

We synthesized HA-DOX via a cost-effective, scalable method using a pH-sensitive hydrazone linker, enabling selective DOX release in the acidic GBM TIME [28,56]. pH-responsive drug release offers an interesting advantage: minimizing off-target toxicity while improving drug bioavailability within the TIME. Although we lack a precise pH value for the GBM resection cavity, we expect to encounter a slightly acidic environment immediately after surgery, due to inflammation, which gradually shifts toward neutrality during healing. The initial acidification could trigger the burst drug release from HA-DOX via linker cleavage; however, as the pH shifts to neutral during healing, HA-DOX remains intact and undergoes uptake by GSCs, thanks to an interaction with the CD44 receptor, before intracellular DOX release occurs in the acidic environment of the lysosome [27,29]. We also note that HA-DOX retained and even improved DOX-mediated ICD effects in SB28 cells. HA may contribute to immune activation by enhancing cellular uptake and stimulating signaling pathways associated with immunogenic responses [25]. When comparing cytotoxicity profiles, DOX and HA-DOX exhibited similar effects in SB28 cells; however, HA-DOX displayed reduced efficacy in SB28-derived GSCs. This discrepancy in drug efficacy could stem from the 3D nature of the GSC model, which exhibits differences in cell density, metabolic functions, cell-surface receptor expression, and proliferation compared to monolayer culture. Supporting this hypothesis, Abbas et al. demonstrated that colorectal cancer cells cultured as 3D spheroids, compared to 2D monolayers, exhibited alterations in cell cycle progression, morphology, nutrient requirements, and overall cellular behavior [57]. Furthermore, Horning et al. observed significantly lower anticancer drug efficacy in their 3D cell model compared to 2D cells [58]. The observed behavior could also result from steric hindrance and the larger size of HA-DOX (15.6 nm) compared to free DOX, which may impair its ability to penetrate dense neurospheres and reach cells in deeper layers. Such limitations remain consistent with the known challenges faced by nanocarriers in vitro [59]. To better understand interactions between HA-DOX and GSCs, we evaluated the colocalization of DOX/HA-DOX with GSC markers in neurosphere cultures. HA-DOX demonstrated greater colocalization than DOX, suggesting improved targeting capacity.

Achieving spatiotemporal control of drug release remains crucial for maximizing therapeutic efficacy while minimizing off-target effects. DOX, while effective, remains clinically limited by dose-dependent toxicity in healthy tissues [60]. To address this, we incorporated HA-DOX into Surgiflo™, modifying its physical properties, notably increasing pore size relative to the blank paste due to HA's high-water retention capacity and its ability to form a hydrated scaffold [61]. The addition of HA-DOX also enhanced paste stability for up to one week, particularly under neutral pH conditions. Cumulative release studies revealed pronounced pH-dependent behavior: we observed full DOX release under acidic conditions but significant DOX retention at neutral pH. We attribute this release pattern to pH-dependent physicochemical interactions between DOX and the gelatin matrix. Both DOX and gelatin remain uncharged at physiological pH, thereby promoting hydrophobic interactions that stabilize the drug within the scaffold and limit its diffusion. In contrast, DOX becomes protonated and positively charged under acidic conditions, reducing its affinity for the gelatin matrix and accelerating release [62,63]. At both pHs, we observed an initial burst release that could provide rapid drug efficacy, followed by sustained release over time that may prove advantageous for treating the GBM resection by targeting and eliminating residual tumor cells, while subsequent sustained delivery maintains therapeutic drug levels for an extended period, helping to prevent recurrence and tumor regrowth by targeting GSCs. These results agree with those reported by Graham-Gurysh et al., who developed an acetalated dextran nanofibrous scaffold tailored for sustained paclitaxel release in the acidic conditions in the resected GBM cavity [64]; therefore, rationally designed release kinetics represent a conceivably efficacious strategy to obtain selective release in the acidic GBM TIME. Moreover, our results agree well with the work of Smith et al. when investigating the efficacy of a paste containing and temozolomide; they observed burst drug release kinetics that may contribute to anti-tumor efficacy. Additionally, the authors note that applying the pastes to the resected cavity limited systemic toxicities [17]. We also tested paste-HA-DOX biocompatibility, revealing no significant differences in markers of neuroinflammation, suggesting that the paste-HA-DOX is fully biocompatible and useful for local brain application since it does not induce chronic neuroinflammation or abnormal glial scarring.

We evaluated the efficacy of paste-HA-DOX using the SB28-based GBM resection model, where we applied the paste to conform to the irregular contours of the resection cavity and maintain close contact with the cavity lining. We observed a significant survival benefit for paste-HA-DOX compared to the untreated group (median survival: 37 vs. 25 days). To further explore the immunological and cellular impacts of paste-based treatment, we examined infiltrating immune cells and GSCs within the tumor and resection cavity 3 days post-treatment. A recent single-cell RNA sequencing study investigated GSC and lineage markers, observing CD44 as the highest expressed marker by mesenchymal-like GSCs and CD133 in oligodendrocyte progenitor cell (OPC)-like GSCs [65]. GSCs do not display uniform distribution within tumors, and their interactions with the microenvironment remain critical to their function [66]. Lu et al. demonstrated that GSCs accumulated in GBM tumor regions containing immune cells, suggesting potential interactions between GSCs and the immune cells of the TIME [66]. These interactions contributed to additional mechanisms of immunosuppression, implying that targeting GSCs may also influence immune cell behavior. Our analysis revealed a trend toward reduced CD44^+^ cell numbers in paste-HA-DOX-treated mice compared with untreated mice, demonstrating HA's targeting capability. These findings agree with related studies that have employed HA for GSC targeting [67]. In GBM models, DOX-loaded chitosan-coated nanoparticles have been employed to target CD44-expressing GSCs; they reported a 6-fold increase in cytotoxicity against GSCs compared to free DOX and a consequent reduction in tumor size in vivo, thereby reinforcing the therapeutic potential of targeting this drug-resistant population [26]. Similarly, CD133+ cells displayed a decreasing trend in number following treatment with paste-HA-DOX in our study. The observed decrease in CD44^+^ and CD133^+^ cell numbers could explain the trend toward increased dendritic cell numbers and the statistically significant increase in CD8^+^ T cells observed after paste-HA-DOX treatment. HA-mediated targeting and DOX-mediated ICD induction might drive the activation of specialized antigen-presenting cells and support CD8^+^ T cell infiltration. The elimination of immune-suppressive GSCs may also contribute; however, HA-DOX might also exert these effects through additional mechanisms independent of its activity on GSCs. Therefore, further studies are required to validate these hypotheses and elucidate the mechanisms driving immune-related changes. GSCs may exhibit resistance to DOX treatment through mechanisms such as active drug efflux via ABC transporters and increased apoptosis resistance [68]. Given the spatial and functional heterogeneity of GSCs across distinct tumor niches, these findings underscore the need for tailored therapeutic strategies that target specific GSC subtypes within their respective microenvironments [66]. Combining our current approach with therapies directed at CD133^+^ cells could represent a promising avenue for achieving more comprehensive tumor eradication.

## 5 Conclusions

We developed an innovative strategy for postoperative GBM treatment using a paste scaffold that provides hemostasis and the sustained release of a GSC and GBM-targeting polymer-drug conjugate (HA-DOX). This approach offers several advantages: (i) targeting GSCs may reduce the risk of disease recurrence and modulate GSC-related immunosuppressive mechanisms in the TIME; (ii) it relies on FDA-approved biomaterials and components that support clinical translation; (iii) it allows for efficient scale-up with a cost-effective strategy and is straightforward for neurosurgeons to handle and apply; and (vi) it generates a ready-to-use formulation capable of conforming to a GBM-resection cavity, bringing it closer to the standard of care. Future studies will involve dose escalation optimization and the design of synergistic combination therapies to enhance the efficacy of this novel approach. Our findings highlight the potential of local therapy at the surgical site to address the critical window between tumor resection and the initiation of systemic chemotherapy.

## CRediT authorship contribution statement

**Giulia Rodella:** Data curation, Formal analysis, Investigation, Methodology, Writing – original draft, Writing – review & editing. **Cristiano Pesce:** Data curation, Formal analysis, Writing – review & editing. **Riccardo Rampado:** Formal analysis. **Mariangela Garofalo:** Formal analysis. **Mingchao Wang:** Data curation, Formal analysis. **Bernard Ucakar:** Data curation, Formal analysis. **Kevin Vanvarenberg:** Data curation, Formal analysis. **Zhanjun Ma:** Data curation, Formal analysis. **Nicolas Joudiou:** Data curation, Formal analysis, Methodology. **Véronique Préat:** Conceptualization, Investigation, Project administration, Supervision, Writing – review & editing. **Bernard Gallez:** Conceptualization, Funding acquisition, Investigation, Project administration, Supervision, Writing – review & editing. **Alessio Malfanti:** Conceptualization, Data curation, Formal analysis, Funding acquisition, Investigation, Methodology, Project administration, Resources, Supervision, Validation, Writing – original draft, Writing – review & editing.

## Declaration of competing interest

The authors declare that they have no known competing financial interests or personal relationships that could have appeared to influence the work reported in this paper.

## Data Availability

Data will be made available on request.
