# Supplementary material for: Localized delivery of hyaluronic acid-doxorubicin from a surgical paste for post-operative glioblastoma treatment
Source: Mater Today Bio. 2026 Apr 22;38:103142. doi: 10.1016/j.mtbio.2026.103142 (PMC13142020; doi:10.1016/j.mtbio.2026.103142)
Supplement: Multimedia component 1 [file mmc1.pdf]

SUPPORTING INFORMATION

Localized Delivery of Hyaluronic Acid-Doxorubicin from a Surgical Paste for Post-operative Glioblastoma Treatment

Giulia Rodella[1,2], Cristiano Pesce[3], Riccardo Rampado[3], Mariangela Garofalo[3], Mingchao Wang[1], Bernard Ucakar[1], Kevin Vanvarenberg[1], Zhanjun Ma[1], Nicolas Joudiou[2], Véronique Préat[1], Bernard Gallez[2*], Alessio Malfanti[1,3*]

[1]UCLouvain, Louvain Drug Research Institute, Advanced Drug Delivery and Biomaterials, Avenue Mounier 73 B1.73.12, 1200, Brussels, Belgium

[2]UCLouvain, Louvain Drug Research Institute, Biomedical Magnetic Resonance, Avenue Mounier 73 B1.73.08, 1200, Brussels, Belgium

[3]Department of Pharmaceutical and Pharmacological Sciences, University of Padova, Via F. Marzolo, 5, 35131 Padova, Italy

*Corresponding authors: bernard.gallez@uclouvain.be; alessio.malfanti@unipd.it

1. Materials and methods

Table S1. List of primers for RT-qPCR

Gene	Forward sequence 5’-3’	Reverse sequence 5’-3’
Rpl19	GAA GGT CAA AGG TGT GTT CA	CCT TGT CTG CCT TCA GCT TGT
Ifnβ	GCA CTG GGT GGA ATG AGA CTA TTG	TTC TGA GGC ATC AAC TGA CAG GTC
Cxcl10	GAC GGT CCG CTG CAA CTG	CTT CCC TAT GGC CCT CAT TCT
Fut4	TCC TTG GGT GGG AAT TAA AGG	CCA GTG TAG ACT TCC GAC AAT AC
Klf4	ACC CAC ACT TGT GAC TAT GC	TTT CTC GCC TGT GTG AGT TC

Table S2. List of antibodies for FACS analysis and immunostaining

Panel	Antibody	Dilutions	Provider and catalogue number
ICD markers	Zombie Aqua™ fixable viability dye	1/1000	BioLegend
	Calreticulin Recombinant Rabbit Monoclonal Ab		BioLegend
	DyLight™ 649 donkey anti-rabbit IgG, IIary Ab		BioLegend
	FITC-labelled Wheat Germ Agglutinin	2 µg/mL	Life Technologies, # W11261
	anti-Calreticulin antibody-APC	1/100	Bio-Techne, NBP 1-47518APC
GSC markers	CD44-AF647	5 µg/mL	BioLegend, 103018
	CD133-AF647	5 µg/mL	BioLegend, 141215
	Nestin-AF647	5 µg/mL	BioLegend, 655107
Biocompatibility	Iba-1 primary rabbit Ab	1/1000	Wako Pure Chemical Industries, 019-19741
	Anti-rabbit APC IIary Ab	1/500	Abcam, ab130805
	GFAP-Cy3	1/300	Sigma Aldrich, C9205
Macrophages and microglia	CD45-Pacific Blue	0.25 µg/10^6 cells	BioLegend, 157211
	CD11b-FITC	0.25 µg/10^6 cells	BioLegend, 101205
	CD3-APC/Cy7	0.25 µg/10^6 cells	BioLegend, 100222
	CD4-PE	0.06 µg/test	BD Biosciences, 557308
	CD8-BV421	0.5 µg/10^6 cells	BioLegend, 100753

	NK1-AF700	0.5 µg/10^6 cells	BioLegend, 156511
	FOXP3-AF488	0.06 µg/test	BD Biosciences, 563487

Protocol tumor imaging

For IVIS imaging, mice were anesthetized with inhaled isoflurane, intraperitoneally injected with D-luciferin (150 mg/kg), and imaged after 15 min. The obtained tumor sizes were expressed as total flux. For MRI imaging, mice were anesthetized with isoflurane and placed on a heating blanket, with temperature and respiration rate monitored. The MRI system was equipped with a 1H quadrature transmit/receive birdcage coil (21 mm inner diameter). Tumors were visualized using a T2-weighted RARE sequence (TR: 2500 ms; TE: 30 ms; Rare factor: 8; number of repetitions: 3; Matrix size: 200 × 200; FOV: 20 × 20 mm^2; slice thickness: 0.5 mm; and total acquisition time: 3 min). Bioluminescence and MRI imaging confirmed tumor presence 12 days after orthotopic SB28 cell grafting. Tumor growth was monitored over time using IVIS and MRI, and the survival rate was assessed (n=6).

2. Results

Table S3. Characterization of HA-DOX according to size, zeta potential, and drug loading (N=3, n=3).

	Size (% number, nm)	Zeta Potential (mV)	Drug Loading (% w/w)
**HA-DOX**	15.9	-21.4	6.71

GSC Marker Gene Expression

The cell medium was removed, and TRIzol™ Reagent (200 µL) and 40 µL chloroform were added to each sample. Cells were centrifuged at 12,000 g for 15 min at 4°C, and the mRNA in the water-soluble phase was precipitated with isopropanol and washed with 75% ethanol. After drying, the mRNA pellet was dissolved in 10 µL endotoxin-free TE buffer, and the total concentration (ng/mL) was measured using a Nanodrop 2000 spectrophotometer (Thermo Scientific, USA). Reverse transcription of mRNA (0.33 µg/µL) was performed using GoScript™ Reverse Transcription Mix, Oligo dT (Promega, WI, USA), and qPCR with GoTaq® qPCR Master Mix (**Table S1** reports the primer sequences).

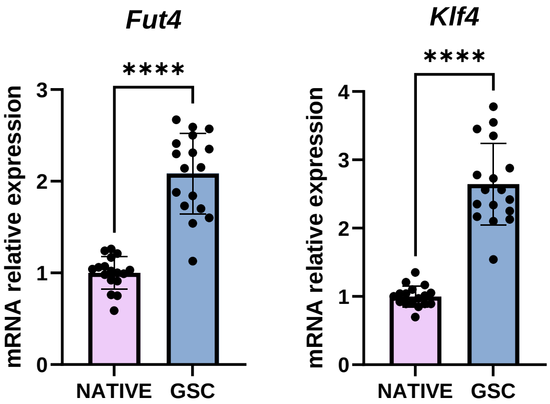

Figure S1. Relative expression of *Fut4* and *Klf4* in native SB28 and GSCs-enriched cells. Relative mRNA expression normalized to *Rpl19* reference gene expression and calculated with the comparative method (2-ΔΔCT). Statistical analyses performed with an unpaired t-test (****p < 0.0001).

Immunohistochemical analysis of unresected and resected SB28 tumors

At day 17, mice were sacrificed, brains were collected, fixed in 4% PFA (v/v in water), then in 10% and 30% sucrose, and finally embedded in OCT and stored at -80°C. Brain tissues were sectioned at a thickness of 12 µm using a Cryostar NX70 cryostat (Thermo Fisher Scientific, Waltham, MA, USA) and mounted onto SuperFrost Plus glass slides. For histological staining, slides were first washed twice with PBST (PBS containing 0.1% Tween 20), then incubated in 3% hydrogen peroxide in methanol for 10 min. After two additional PBST washes, sections were blocked for 1 h in blocking solution containing 10% goat serum, 2% BSA, and 0.2% Triton X-100. Primary antibodies against Ki67 (1:200) or CD44 (1:200), diluted in the blocking solution, were applied to the sections. After incubation, slides were treated with an anti-rabbit HRP-conjugated secondary antibody for 40 min. Excess antibody was removed, and the sections were developed using DAB substrate for 10 min. Slides were then rinsed under running tap water for 10 min. Counterstaining was performed with hematoxylin for 45 s, followed by a 5-min rinse under tap water. Slides were briefly dipped in lithium carbonate for 5 s, then rinsed under tap water for 5 min. Dehydration was performed by three consecutive 2-min incubations in isopropanol, followed by three 2-min incubations in Histosave. Finally, slides were mounted using DPX mounting medium.

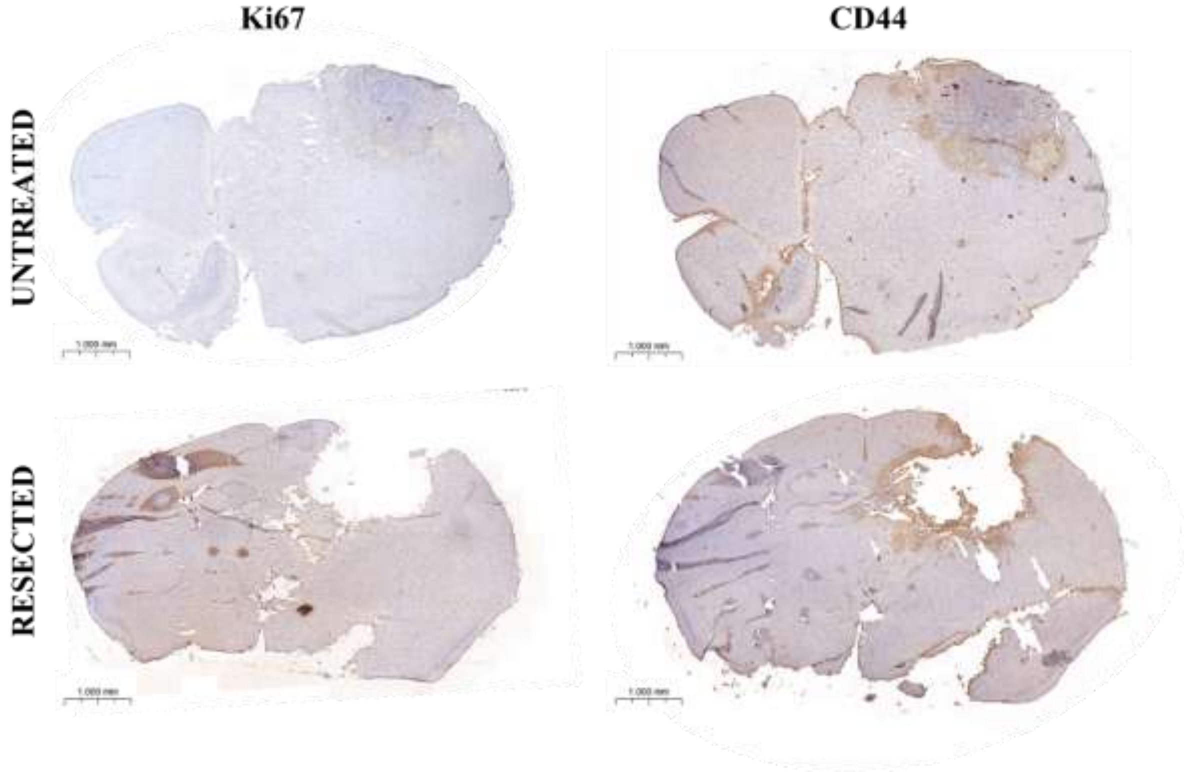

Figure S2. Representative immunohistochemistry images of unresected and resected SB28 tumors for Ki67 and CD44 staining.

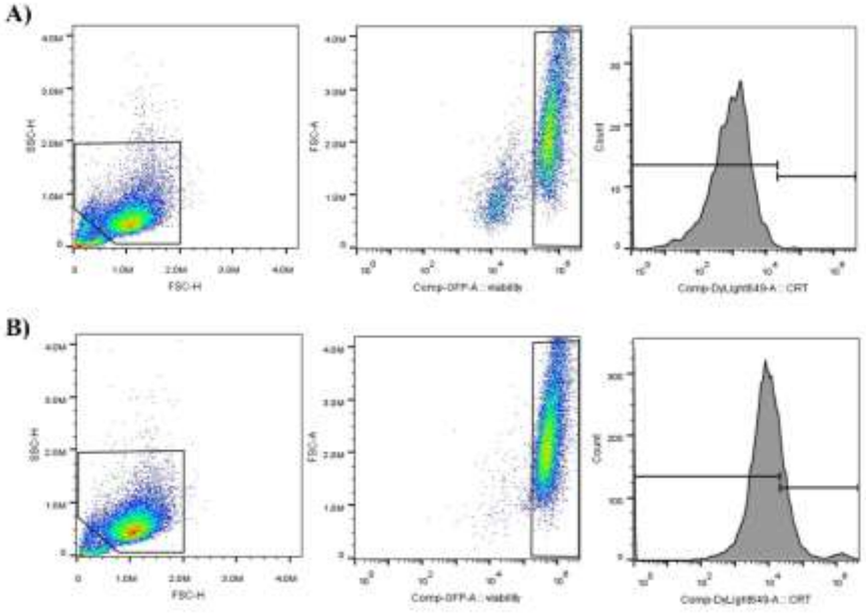

Figure S3. **Gating strategy for analysis of CRT exposure via FACS analysis of SB28 cells**. Cells were selected from a forward scatter height (FSC-H) vs side scatter height (SSC-H) dot plot, subsequently, living cells were selected according to GFP-positive cells (viable SB28 cells expressing GFP), and finally, CRT⁺ cells were selected in comparison to the unstained sample **(A)**, which was set as a negative control, to identify the CRT⁺ cells in the stained sample **(B).**

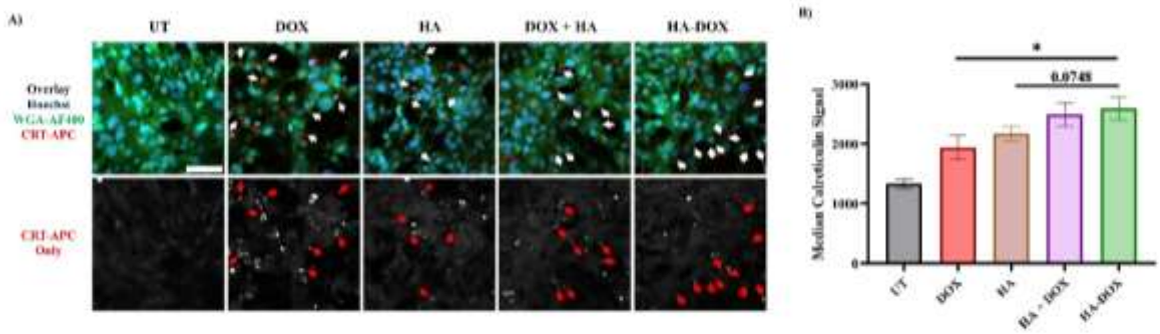

Figure S4. Immunogenic cell death detection via CRT exposure. A) Representative confocal images of SB28 cells treated with DOX, HA, HA+DOX, or HA-DOX at 0.1 µM (DOX equiv.) for 24 h. CRT staining in red; cell membranes stained with WGA-AF400; nuclei stained with Hoechst (blue). 40X magnification. **B)** Median CRT signal (n=3). Statistical analyses performed with an unpaired t-test (*p < 0.05).

Intracellular localization of DOX and HA-DOX

SB28 GSCs (2 × 10^5 cells/well) were seeded in round-bottom plates with ultra-low attachment 96-well plates, centrifuged 1 min at 300 g to favor cell sedimentation, and incubated for 24 h with 5 µM DOX and HA-DOX (DOX equiv.) in GSC-enriched media. GSCs were then collected, washed three times with

PBS, and fixed with 4% PFA (v/v in water) for 1 h at room temperature. Afterwards, GSCs were washed three times with PBS, and Triton 0.2% was added for 10 min at room temperature. Samples were stained with Alexa Fluor® 647 anti-mouse/human CD44 antibody (5 µg/mL) or Alexa Fluor® 647 anti-mouse Nestin antibody (5 µg/mL) for 1 h in the dark at 4°C. Finally, cells were stained with DAPI (dilution 1:1000) for 1 h at 4°C. After washing, each neurosphere was transferred to an uncoated µ-Slide 15 well 3D (ibidi Gräfelfing, Germany), the excess of PBS was removed, and 50 µL HIGHDEF® IHC fluoromount (Enzo Life Sciences, Belgium) was added. Confocal microscopy was performed with a Cell Observer spinning-disk confocal microscope (Zeiss, Oberkochen, Germany) using a 40X objective, and Z-stack images were captured. DAPI was detected at λex: 405 nm, DOX at λex: 555nm, and CD44/Nestin at λex: 633 nm. Images were analyzed with ZEN Blue software, and colocalization quantification was performed with ImageJ software (Bethesda, MD, USA).

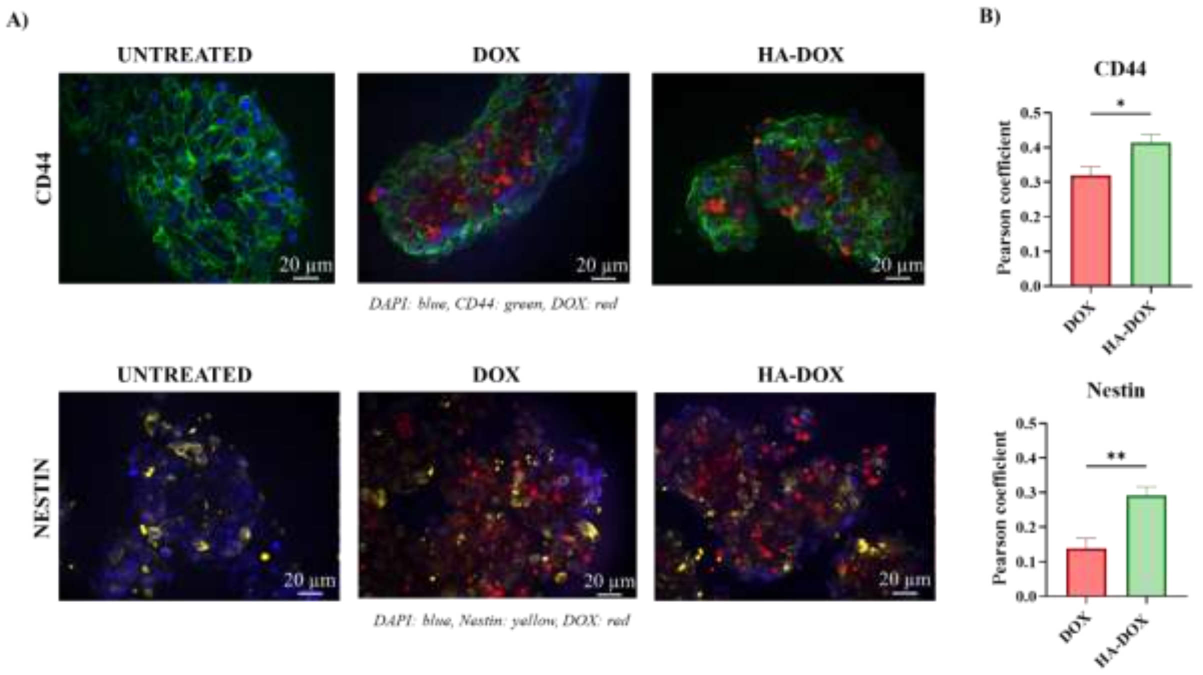

Figure S5. Intracellular localization of DOX and HA-DOX following 24-h treatment. A) Representative confocal microscopy images of CD44 (green) and Nestin (yellow) colocalization with DOX/HA-DOX (red) in GSCs following 24 h treatment with 5 µM DOX or HA-DOX. Nuclei are stained with DAPI (blue). Objective 40x; scale bar = 20 µm. **B)** Pearson coefficient values of DOX or HA-DOX colocalization with CD44 and Nestin (n=3). Statistical analyses performed with an unpaired t-test (*p < 0.05; **p < 0.01).

A)

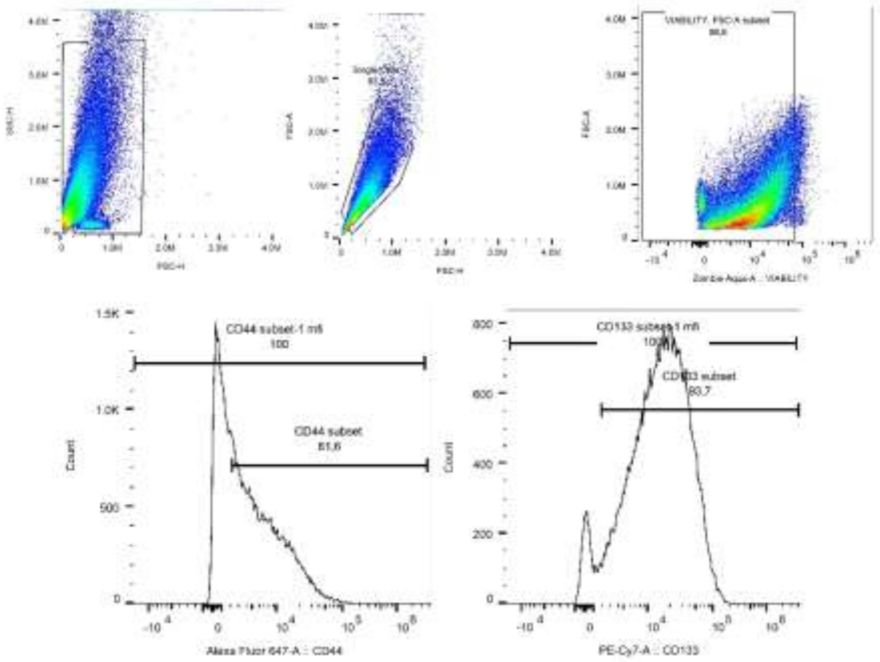
CD44 subset-1 mfi
100
CD44 subset
81,6
Count
Alexa Fluor 647-A :: CD44
CD133 subset
83,7
PE-Cy7-A :: CD133

B)

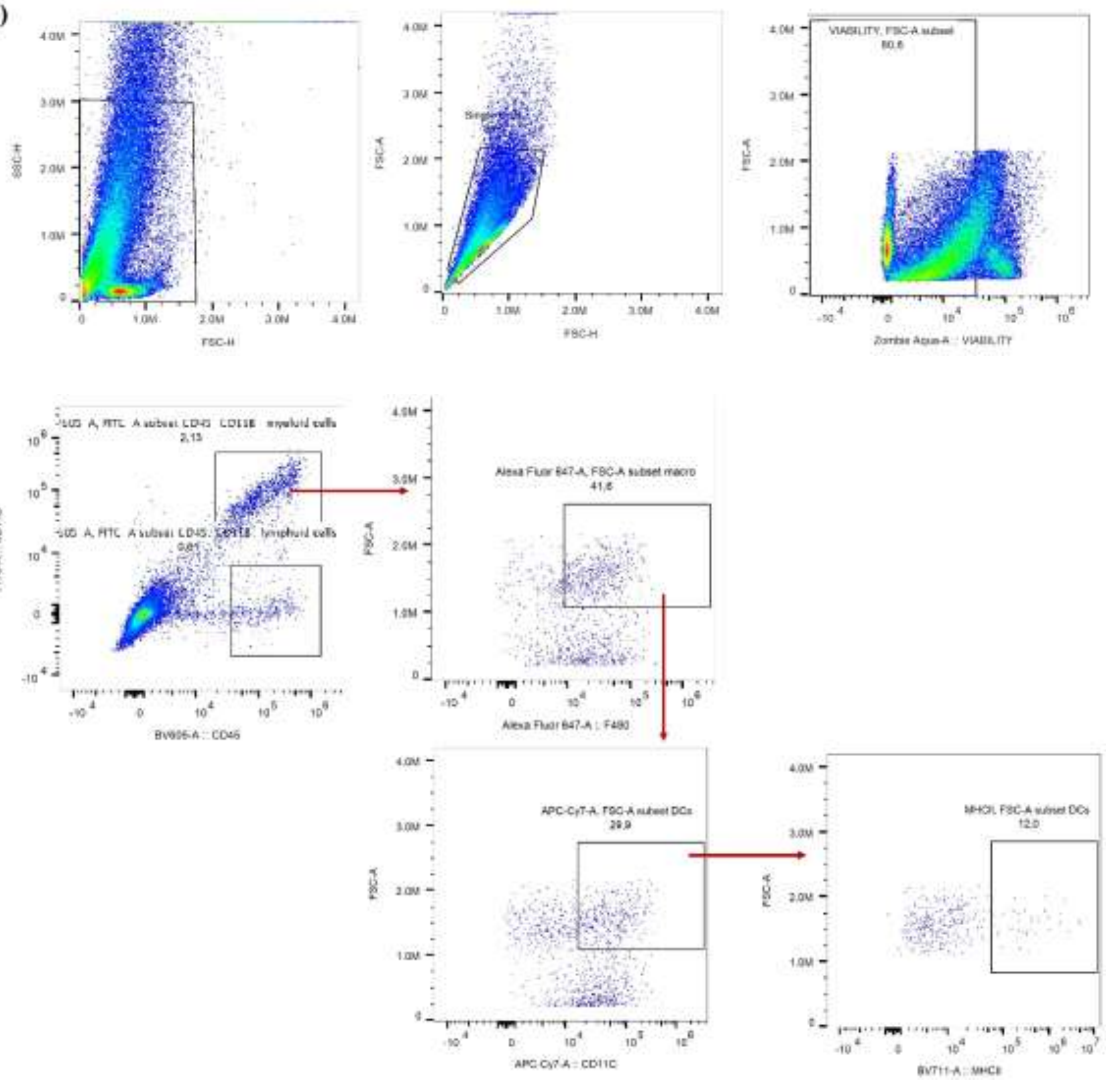
SSC-H
FSC-H
FSC-A
VIABILITY, FSC-A subset
80,6
Zombie Aqua-A :: VIABILITY
FITC-A :: CD11B
BV605-A :: CD45
Alexa Fluor 647-A, FSC-A subset macro
41,6
Alexa Fluor 647-A :: F480
APC-Cy7-A, FSC-A subset DCs
29,9
APC Cy7-A :: CD11C
MHCII, FSC-A subset DCs
12,0
BV711-A :: MHCII

C)

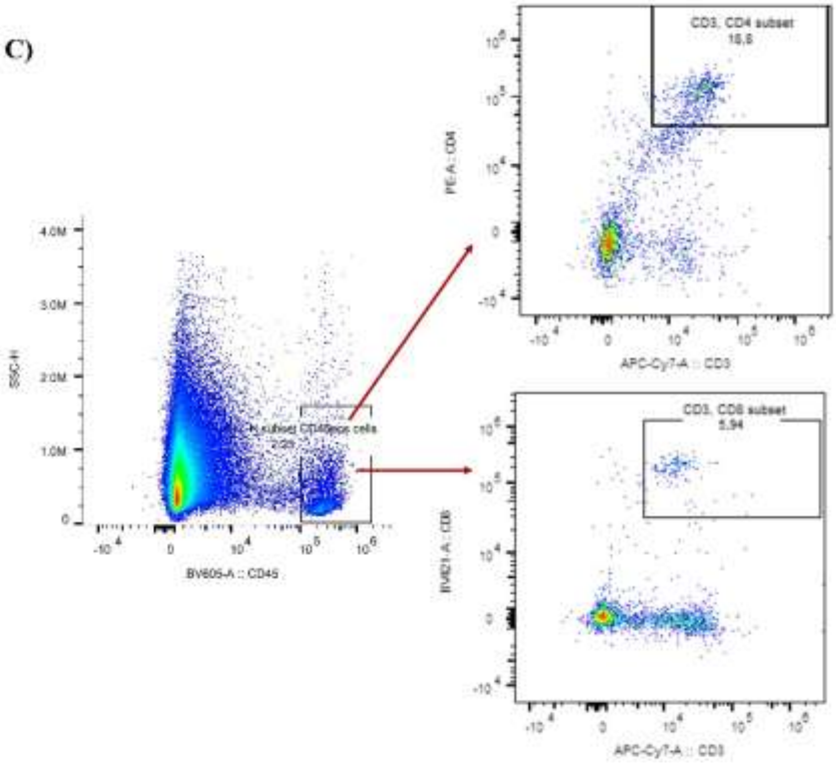

Figure S6. Gating strategy cell analysis for the TIME and GSCs. Cells were selected from the FSC-H vs. SSC-H dot plot, and subsequently, single cells were selected from the side scatter area (SSC-A) plot vs. SSC-H plot; then, viable cells were considered according to Zombie Aqua staining. **A)** CD44+ GSCs and CD133+ GSCs: histogram plots were used to identify the positive cell population; **B)** Dendritic and macrophages (CD45+CD11b+ represented myeloid cells) - from this population: F4/80+ cells were macrophages and CD11c+/MHCII+ were dendritic cells; and **C)** T cells - from CD45+/CD3+ cells among viable cells, CD4+ T cells and CD8+ T cells. In the CD4+ population, Treg cells expressed FoxP3.

A)

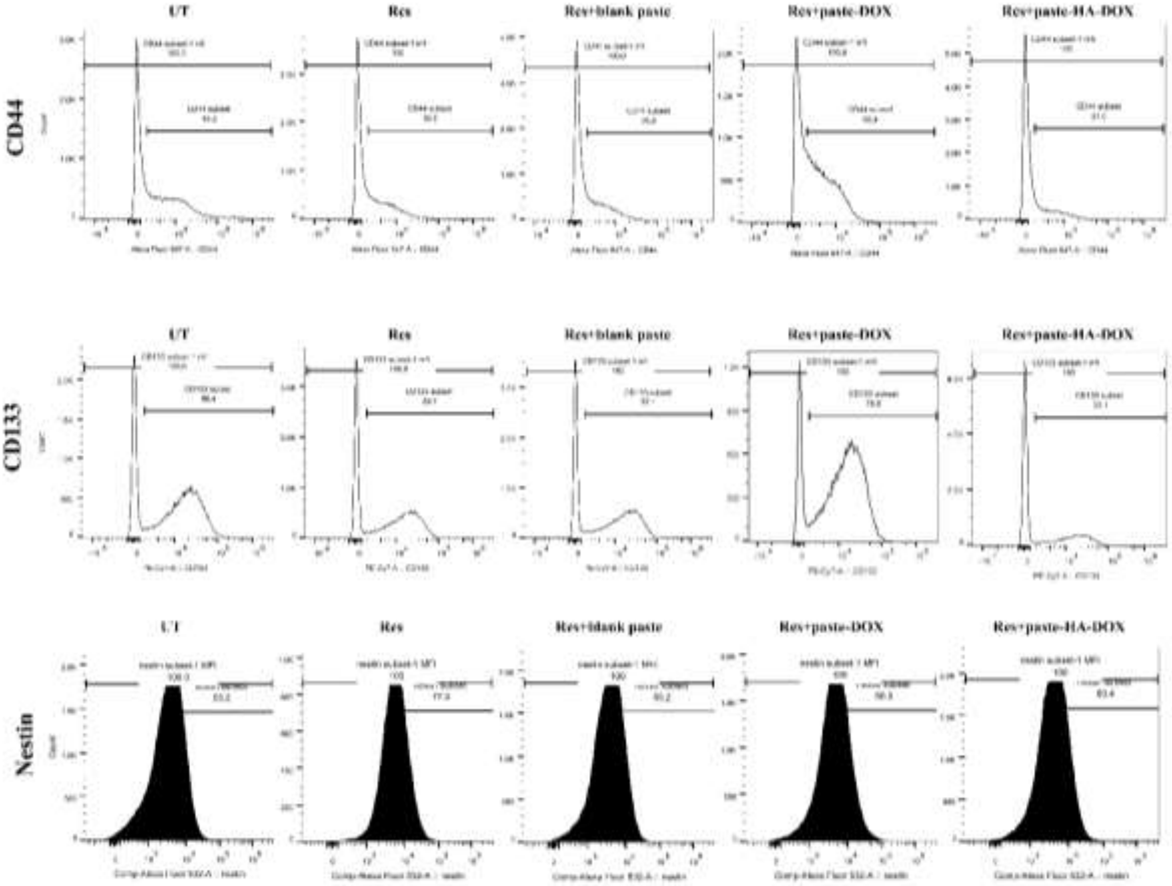
U/T
Res
Res+blank paste
Res+paste-DOX
Res+paste-HA-DOX
CD44
CD133
Nestin

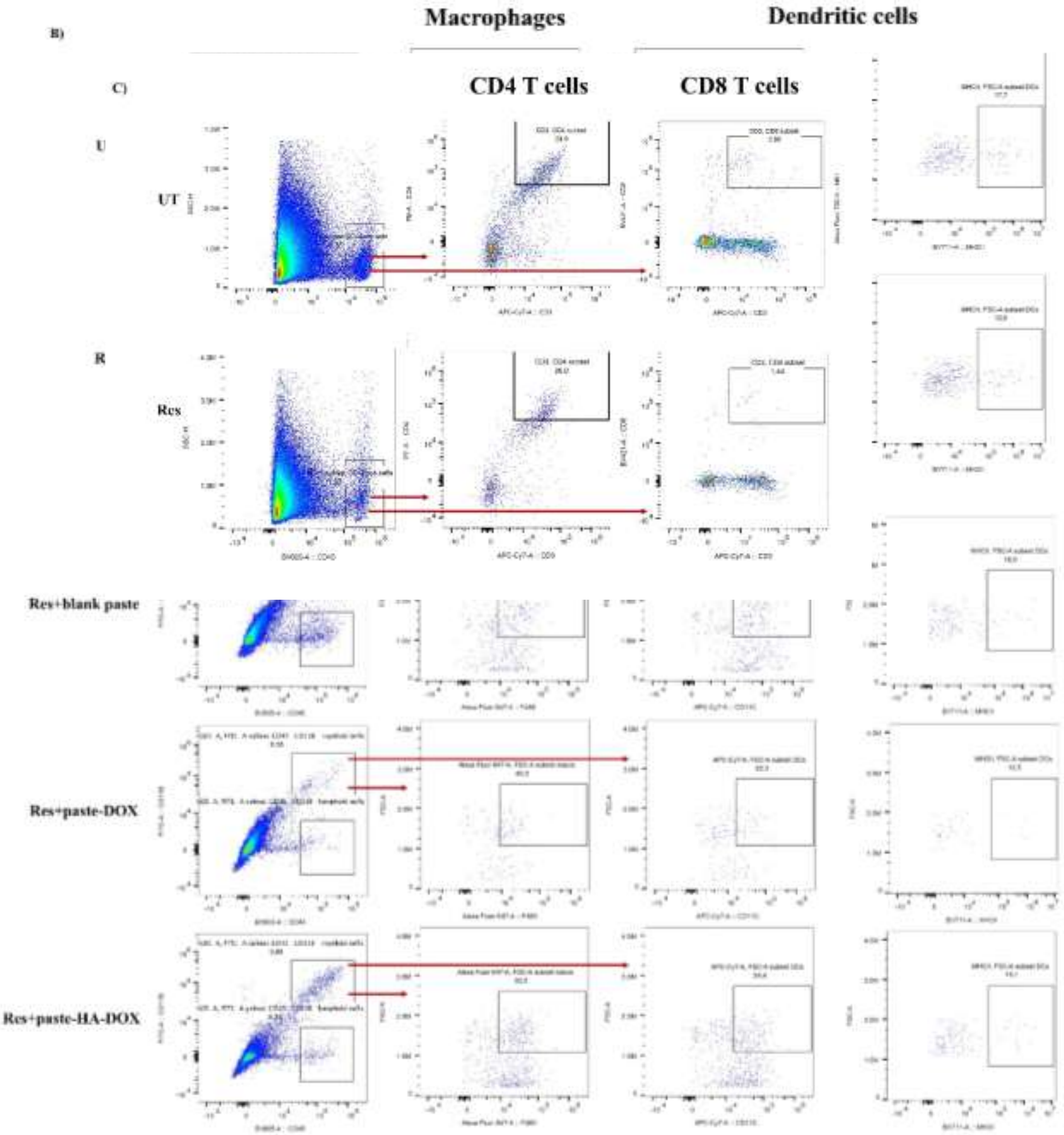
B)
Macrophages
Dendritic cells
C)
CD4 T cells
CD8 T cells
U
UT
R
Res
Res+blank paste
Res+paste-DOX
Res+paste-HA-DOX

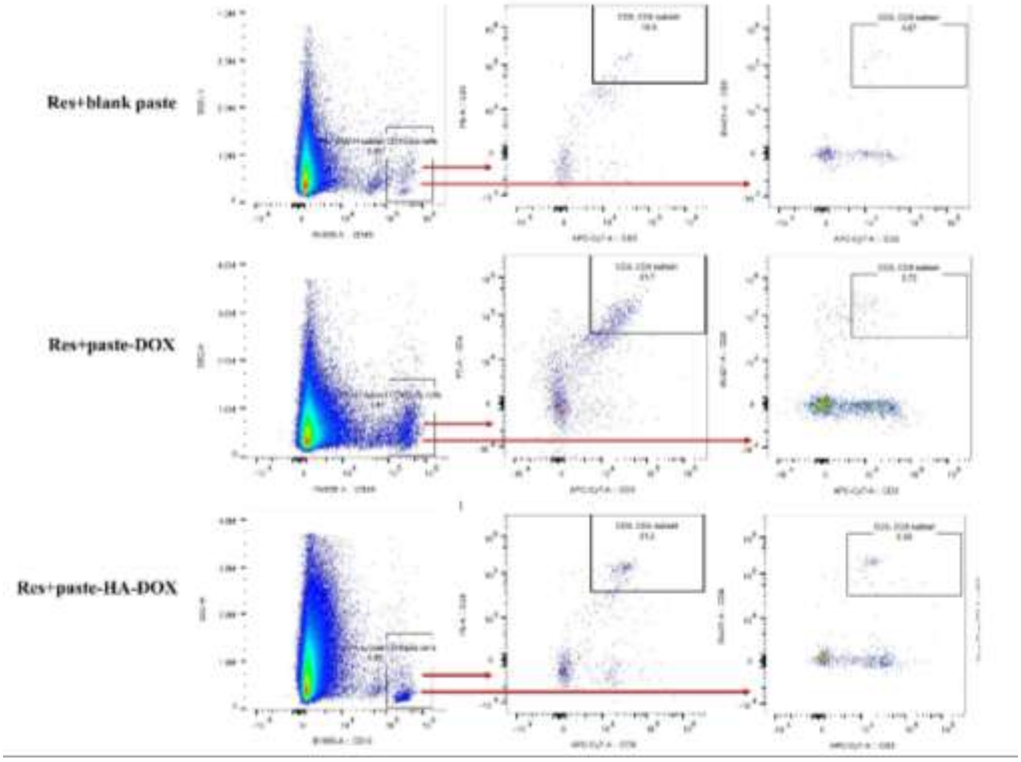

Figure S7. Flow cytometry charts per group. **A)** CD44, CD133 and nestin; **B)** macrophages and DCs; **C)** CD8 and CD4 T cells

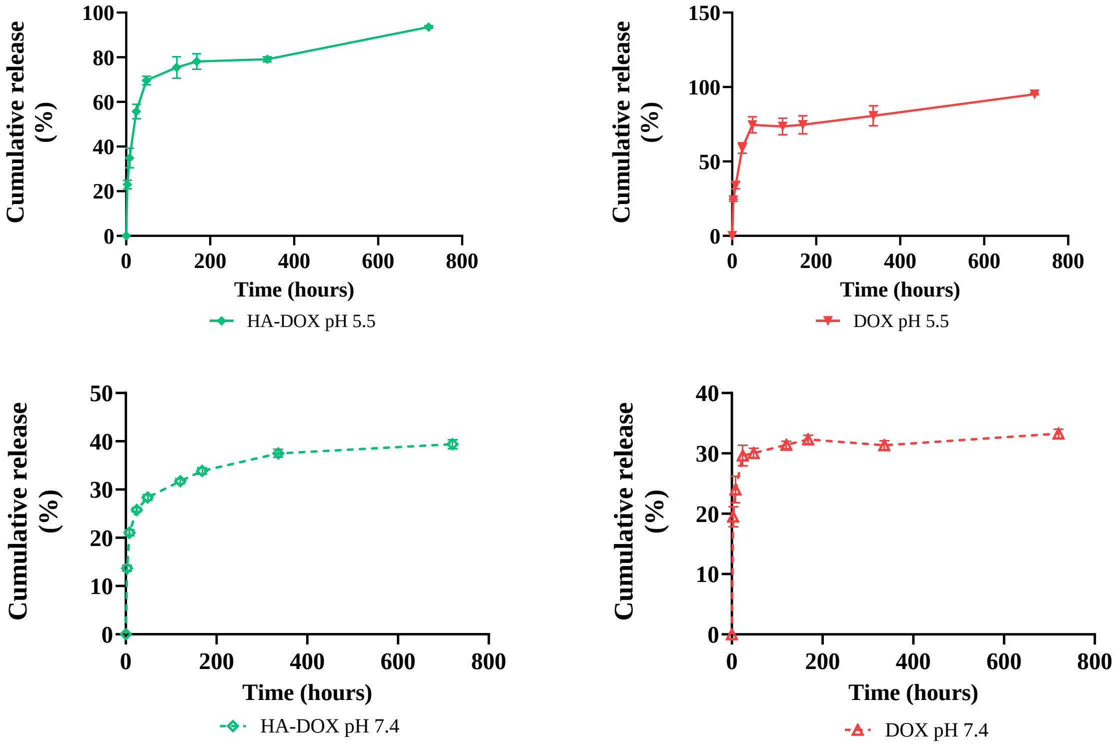

Figure S8. Single plots of cumulative drug release from paste-DOX and paste HA-DOX over time in vitro following incubation in CSF-like medium at pH 5.5 or 7.4.

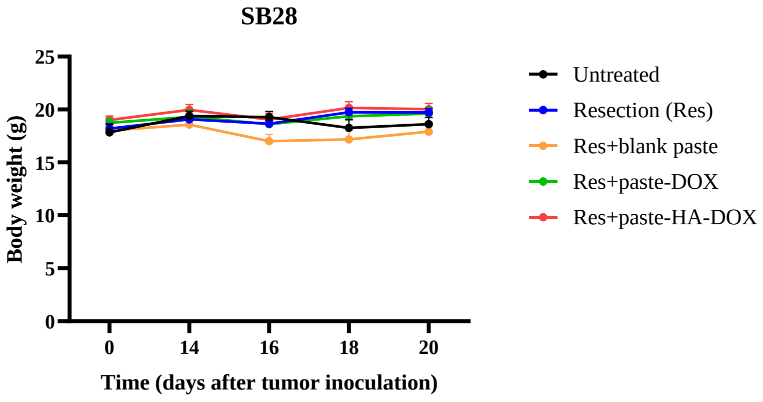

Figure S9. Body weight curves for untreated (n=5), resected (Res) (n=5), Res+blank paste (n=6), Res+paste-DOX (n=5), and Res+paste-HA-DOX (n=7) mice.

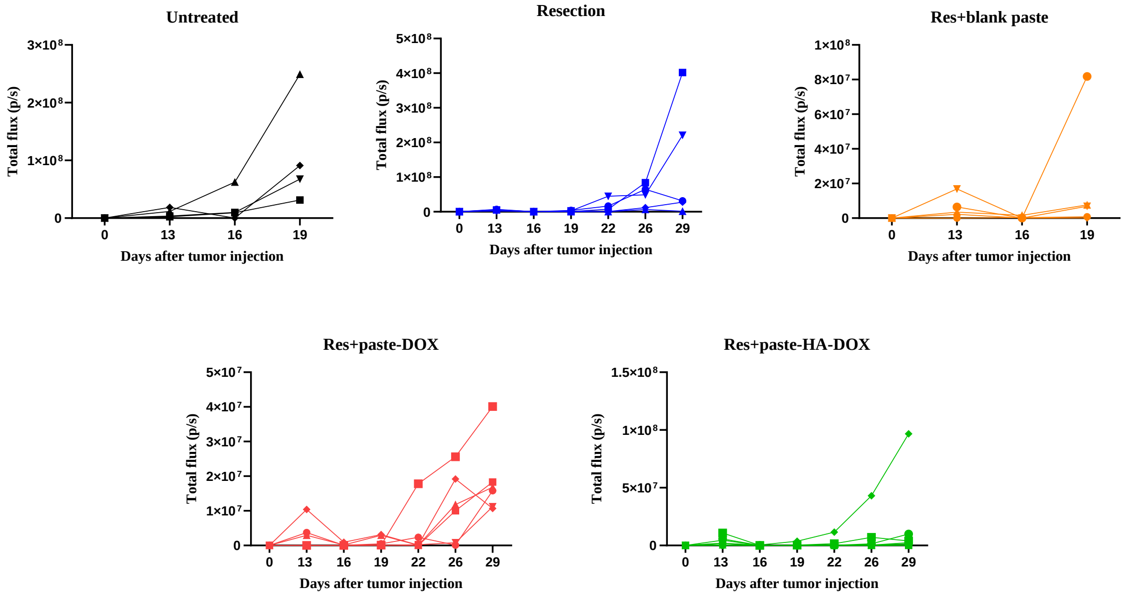

Figure S10. Tumor volume growth curves for individual mice orthotopically grafted with SB28 in each treatment group.
